# The E3 SUMO ligase PIASγ is a novel interaction partner regulating the activity of diabetes associated hepatocyte nuclear factor-1α

**DOI:** 10.1038/s41598-018-29448-w

**Published:** 2018-08-24

**Authors:** Alba Kaci, Magdalena Keindl, Marie H. Solheim, Pål R. Njølstad, Lise Bjørkhaug, Ingvild Aukrust

**Affiliations:** 1 KG Jebsen Center for Diabetes Research, Department of Clinical Science, University of Bergen, 5021 Bergen, Norway; 2 Department of Medical Genetics, Haukeland University Hospital, 5021 Bergen, Norway; 3 Department of Pediatrics and Adolescents, Haukeland University Hospital, 5021 Bergen, Norway; 4 Department of Biomedical Laboratory Sciences and Chemical Engineering, Western Norway University of Applied Sciences, 5063 Bergen, Norway

## Abstract

The transcription factor hepatocyte nuclear factor-1α (HNF-1A) is involved in normal pancreas development and function. Rare variants in the *HNF1A* gene can cause monogenic diabetes, while common variants confer type 2 diabetes risk. The precise mechanisms for regulation of HNF-1A, including the role and function of post-translational modifications, are still largely unknown. Here, we present the first evidence for HNF-1A being a substrate of SUMOylation *in cellulo* and identify two lysine (K) residues (K205 and K273) as SUMOylation sites. Overexpression of protein inhibitor of activated STAT (PIASγ) represses the transcriptional activity of HNF-1A and is dependent on simultaneous HNF-1A SUMOylation at K205 and K273. Moreover, PIASγ is a novel HNF-1A interaction partner whose expression leads to translocation of HNF-1A to the nuclear periphery. Thus, our findings support that the E3 SUMO ligase PIASγ regulates HNF-1A SUMOylation with functional implications, representing new targets for drug development and precision medicine in diabetes.

## Introduction

Hepatocyte nuclear factor-1α (HNF-1A) is a transcription factor encoded by the *HNF1A* gene, which regulates several pancreas and liver specific genes, and plays a role in pancreas/liver development and function^1^. In pancreatic β-cells, HNF-1A is part of a regulatory circuit involving other transcription factors like the pancreatic duodenal homeobox-1 (PDX-1), the hepatocyte nuclear factor-4 alpha (HNF-4A) and-1 beta (HNF-1B), which are important for normal glucose-induced insulin secretion^1,2^. In mice, loss of *Hnf1a* function results in multiple metabolic abnormalities including defects in pancreatic β-cell glucose sensing, hypercholesterolemia and aberrant expression of genes involved in pancreatic islet development and metabolism^3^, further illustrating the important role of HNF-1A in controlling pancreatic-islet β-cell function^4^. Although rare variants in *HNF1A* are primarily associated with a monogenic form of diabetes known as Maturity-Onset Diabetes of the Young (MODY3; HNF1A-MODY, OMIM #600496)^5^, common *HNF1A* variants represent risk factors for type 2 diabetes^6–8^.

In its active form, HNF-1A functions as a homodimer or heterodimer (with HNF-1B), and both complexes are stabilized by the dimerization cofactor DCoH^9^. Other cofactors involved in HNF-1A transcriptional regulation are the CREB-binding protein (CBP)^10^, the CBP-associated factor (P/CAF)^10^, and the high mobility group protein-B1^11^, exerting a positive effect on HNF-1A transactivation function. Post-translational modifications (PTMs) are known to regulate HNF-1A. Phosphorylation of HNF-1A by the ATM kinase, results in enhanced HNF-1A transcriptional activity^12^, and ubiquitin-induced proteasomal degradation is a mechanism for its intracellular clearance^13^. Still, the precise mechanisms for transcriptional regulation of HNF-1A, including the role and function related to PTMs, are so far largely unknown.

SUMOylation represents a highly dynamic and reversible ATP-consuming PTM process of proteins, involving a cascade of different proteins/enzymes including an E1 (activating enzyme), E2 (conjugating enzyme), an E3 SUMO ligase, and is reversed by the SUMO-specific proteases of the SENP family^14^. In pancreatic β-cells, SUMOylation regulates the function of key proteins involved in insulin secretion, including transcriptions factors like MafA^15^ and PDX-1^16^, the glucose sensor glucokinase^17^, and the voltage-dependent K(+) (Kv) channel Kv2.1^18^. Thus, targeting the SUMOylation cascade has been proposed as a relevant diabetes treatment approach^19^.

Here, we report the first evidence that HNF-1A is modified by SUMO-3 in *cellulo* and that its level of SUMOylation is enhanced by the action of the E3 SUMO ligase; protein inhibitor of activated STAT (PIASγ). Although the presence of SUMO-3 and PIASγ did not affect the nuclear level of HNF-1A protein, or its DNA binding ability, PIASγ repressed the transcriptional activity of HNF-1A. Furthermore, PIASγ was demonstrated to interact with HNF-1A, and sequestrated HNF-1A in the nuclear periphery, hence inducing its transcriptional repression of the HNF-1A target genes *Ace2* and *Slc2a2*. In this study, we report for the first time that both SUMOylation of HNF-1A by SUMO-3 and its interaction with PIASγ are novel mechanisms regulating the nuclear localization and hence transcriptional activity of HNF-1A.

## Results

### HNF-1A is a target for SUMO-3 modification

**Figure 1:**
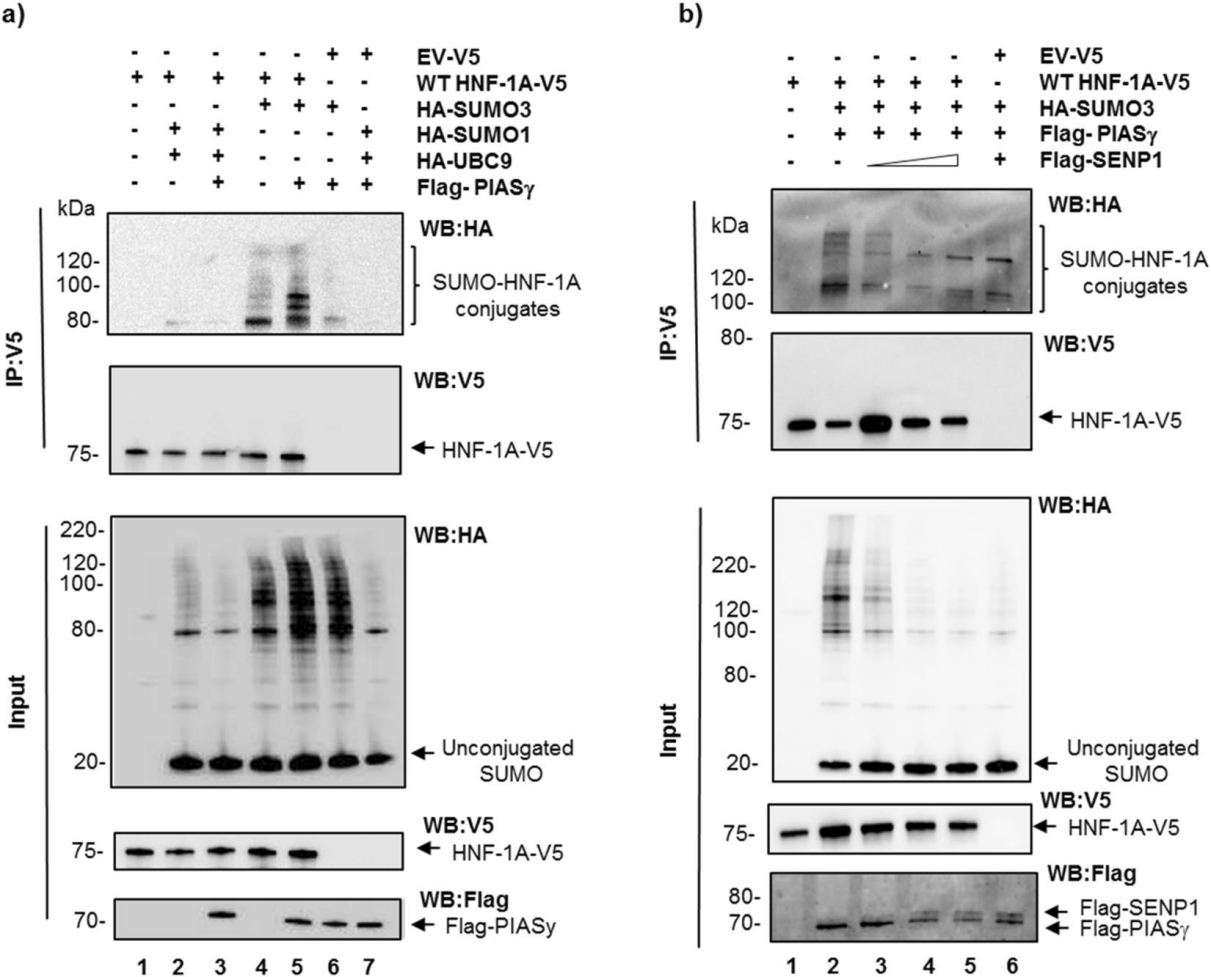
HNF-1A is SUMOylated by SUMO-3 and PIASγ in HEK293 cells. (**a**) SUMOylation of HNF-1A analyzed in HEK293 cells transiently transfected with V5-tagged HNF-1A or empty vector together with HA-tagged SUMO-1 or SUMO-3, HA-tagged UBC9 and/or Flag-tagged PIASγ. Cells were lysed in the presence of N-Ethylmaleimide and protease inhibitors, and subjected to immunoprecipitation using anti-V5 antibody. The precipitate (upper half) and 10 µg input (lower half) were separated by SDS-PAGE and immunoblotting using anti-HA, anti-V5 and anti-Flag antibodies. Full-length blots are presented in Supplementary Fig. S3. This experiment was replicated in three independent experimental days (n = 3). (**b**) De-SUMOylation of HNF-1A by SENP-1. HEK293 cells were transiently transfected with V5-tagged HNF1A or empty vector together with HA-tagged SUMO3, Flag-tagged PIASγ, and increasing amounts of Flag-tagged SENP-1 (0.25–0.75 µg). Samples isolated by V5-immunoprecipitation were analyzed by SDS-PAGE and immunoblotting as indicated above. Full-length blots are presented in Supplementary Fig. S4. This experiment was replicated in two independent experimental days (n = 2).

To investigate whether HNF-1A is a target of SUMOylation, we overexpressed HEK293 cells with HNF-1A (V5-tagged), SUMO-1 or SUMO-3 (HA-tagged), and in the presence or absence of the E2 enzyme Ubc9 (HA-tagged) or the E3 SUMO ligase, PIASγ (Flag-tagged). HNF-1A was further isolated by V5-tag immunoprecipitation and analyzed by SDS-PAGE and immunoblotting using tag-specific (HA and V5) antibodies (Fig. 1a). In the immunoprecipitated samples analyzed by anti-HA antibody, several higher molecular-mass bands ranging from ∼90–200 kDa, corresponding to HA-SUMO-conjugated HNF-1A was observed in cells co-transfected with SUMO-3 (Fig. 1a). Notably, HNF-1A seems to be preferentially modified by SUMO-3, versus SUMO-1, as only one faint high molecular-mass band could be observed in cells co-transfected with SUMO-1. Moreover, the presence of PIASγ in the co-transfection further enhanced the efficiency of SUMO-3 conjugation of HNF-1A, but seemed not to affect SUMO-1 conjugation. No high molecular-mass bands could be observed in control samples with HNF-1A alone or in samples transfected with empty vector and the SUMOylation machinery. Furthermore, in the input samples (Fig. 1a) we could also observe a clear difference in the level of conjugation of SUMO-1 versus SUMO-3 to proteins in general, where the latter seemed to be more frequently conjugated to proteins in HEK293 cells. Moreover, the presence of PIASγ further increased the level of total SUMO-3 conjugated proteins (Fig. 1a), but seemed to have no effect on SUMO-1 conjugation. Next, to further verify that the high molecular-mass bands, seen in the presence of SUMO-3 and PIASγ, indeed correspond to HNF-1A SUMOylated forms, we performed an analogous immunoprecipitation experiment in the presence of the SUMO-specific protease SENP-1. As shown in Fig. 1b, overexpression by increasing amounts of SENP-1 reduced the level of SUMO-3 conjugated HNF-1A bands confirming that HNF-1A is a target for SUMO-3 modification. These findings were further supported by our results showing that endogenous mouse Hnf-1a from MIN6 cells was modified by exogenous SUMO-3, and the level of SUMO-3 modification increased by co-transfection of PIASγ and reduced by the presence of SENP-1 (Supplementary Fig. S1).

### K205 and K273 are SUMO-3 target residues in HNF-1A

**Figure 2:**
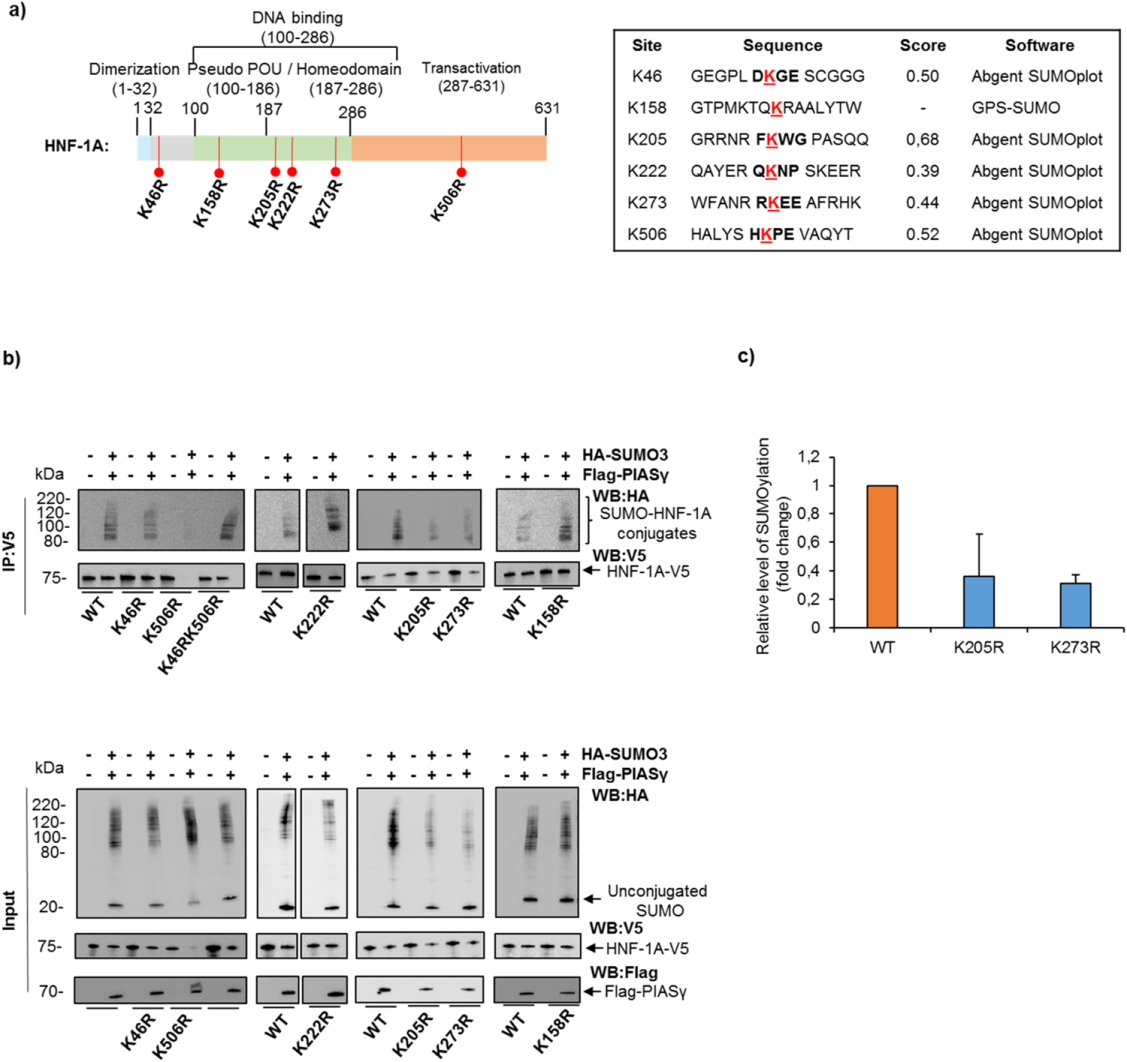
K205 and K273 are SUMOylation sites in HNF-1A. (**a**) Schematic overview of the HNF-1A protein highlighting the lysine residues predicted as SUMOylation sites using the *in silico* prediction programs SUMOplot and GPS-SUMO. The predicted motif for covalent SUMO attachment is displayed with the lysine residue underlined in red. (**b**) Site directed mutagenesis of HNF-1A demonstrated loss of SUMOylation upon substitution lysine (K) with arginine (R), at residues K205, K273 and K506 in HEK293 cells. Cells were transfected with V5-tagged HNF-1A (WT or mutants) together with HA-tagged SUMO-3 and Flag-tagged PIASγ. Lysates were collected in the presence of NEM and protease inhibitors and subjected to immunoprecipitation using anti-V5 antibody. The precipitates were separated by SDS-PAGE and immunoblotting using anti-HA and anti-V5 antibodies. Full-length blots are presented in Supplementary Fig. S5. This experiment was replicated in three independent experimental days (n = 3). (**c**) Quantification of high molecular SUMOylated bands by densiometric analysis is presented as relative fold change compared to WT alone (set to 1). Each bar represents a mean of three independent experiments ± SD (n = 3).

SUMOylation usually occurs within a specific SUMO consensus motif described as ψKXE, in which ψ is a large hydrophobic residue, X is any amino acid and K (Lysine) is the SUMO conjugation site^14^. However, SUMOylation at lysine residues outside this motif has also been described^14,20,21^. Using the *in silico* prediction programs SUMOplot (Abgent) and GPS-SUMO (The Cuckoo Workgroup) several lysine residues (K46, K158, K205, K222, K273 and K506) were predicted as possible candidates for SUMO-conjugation in HNF-1A, with varying scores (Fig. 2a). However, none of the predicted lysine residues were within a SUMO consensus motif. All predicted lysine mutants were generated and their effects on SUMOylation of HNF-1A were examined in HEK293 cells. K205R, K273R and K506R showed substantial reduction in the intensity of the higher molecular mass SUMOylated HNF-1A bands compared to WT (Fig. 2b,c), indicating that these three residues represent HNF-1A SUMOylation sites and were therefore chosen for further investigations. Interestingly, overexpression of SUMO-3 and PIASγ seemed to reduce the protein level of unmodified WT HNF-1A and all the mutants, observed in input samples (Fig. 2b), and most dramatically for the K506R mutant, whose unmodified band was almost undetectable when overexpressed with SUMO-3 and PIASγ. Due to the instability of the K506R mutant in the presence of the SUMOylation machinery, it was difficult to conclude whether K506 is a true SUMOylation site. Since both the SUMO-site mutants, K205R or K273R, showed some degree of SUMOylation, we investigated the level of SUMO-conjugation of a double mutant (K205RK273R) and a triple mutant (K205RK273RK506R) in HEK293 cells (Supplementary Fig. S2). Both mutants showed a reduced level of higher molecular mass SUMOylated HNF-1A bands compared to WT HNF-1A, but both mutants retained some degree of SUMOylation.

### HNF-1A transactivation is repressed by PIASγ in MIN6 cells and requires simultaneous HNF-1A SUMOylation at K205 and K273

**Figure 3:**
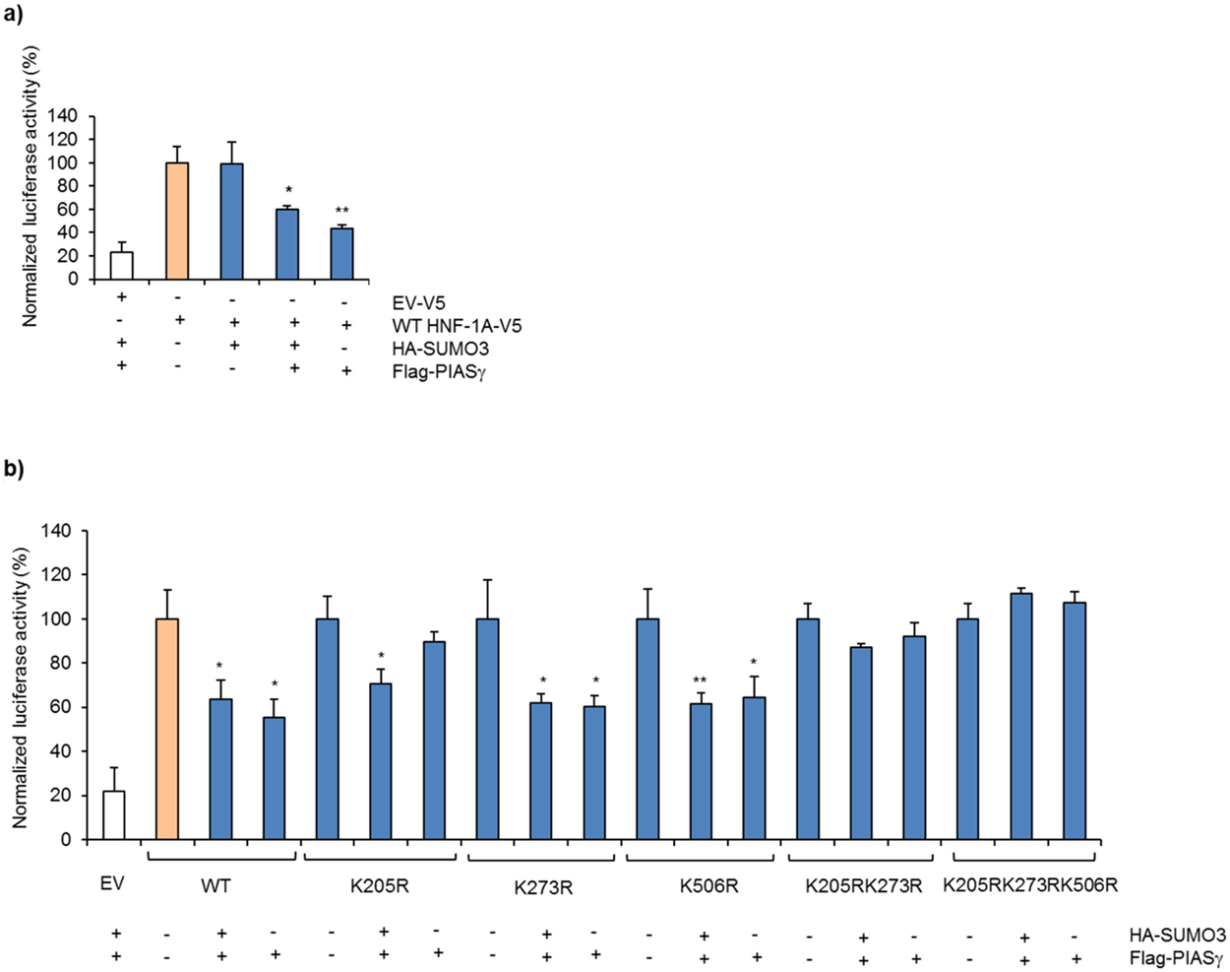
HNF-1A transactivation of a rat albumin promoter is repressed by SUMO-3 and PIASγ. (**a**,**b**) Transactivation analysis of HNF-1A in MIN6 cells transiently transfected with WT HNF-1A or mutants K205R, K273R, K506R, K205RK273R and K205RK273RK506R in the presence/absence of plasmids encoding SUMO-3, and/or PIASγ, as indicated in the figure, and together with the reporter plasmids pGL3-RA and pRL-SV40. *Firefly* luciferase activity was normalized to *Renilla* activity (control reporter) and empty vector was used as negative control. Each bar (**a**,**b**) represents the mean of nine readings ± SD; three parallel readings were conducted on each of three experimental days (n = 3). Measurements are given in fold activity relative to WT. *indicates p < 0.05, **indicates p < 0.001. EV = empty vector.

SUMOylation has been shown to affect the activity of many transcription factors, acting in most cases as a repressor of target gene transactivation^22,23^. We firstly assessed the presence and effect of SUMO-3 and PIASγ overexpression on HNF-1A transactivation of a *Firefly* luciferase reporter gene via its regulation of a rat albumin promoter in transfected MIN6 cells Co-expression of SUMO-3 and PIASγ, significantly reduced the activity of HNF-1A to around 60%, compared to cells transfected with HNF-1A alone (100%) (Fig. 3a). While co-expression of SUMO-3 alone did not affect the transcriptional activity of HNF-1A, co-transfection with PIASγ alone demonstrated the strongest inhibitory effect and reduced HNF-1A transactivation even more than when overexpressed together with SUMO-3 (~40% and ~60%, respectively, compared to HNF-1A alone of 100%) (Fig. 3a). Next, to investigate whether loss of potential SUMO-target lysine residues would obviate the PIASγ-mediated repression, the effect of SUMO-3 and/or PIASγ on the transcriptional activity of single mutants (K205R, K273R and K506R), double mutant (K205RK273R) and triple mutant (K205RK273RK506R) was assessed (Fig. 3b). The basal activities of all mutants were per se significantly lower compared to WT HNF-1A (Supplementary Fig. S3). Similarly to WT, the activity of the single mutants K205R, K273R and K506R was also significantly reduced (around 60–70%) in the presence of both SUMO-3 and PIASγ (Fig. 3b), supporting that the transcriptional repression does not require HNF-1A SUMOylation at one single lysine residue. By contrast, the transactivation activity of the double and triple mutants, K205RK273R and K205RK273K506R, were not reduced by either SUMO-3 and PIASγ, or PIASγ alone, indicating that lack of SUMOylation at these sites inhibit repression of HNF-1A transactivation (Fig. 3b).

### SUMOylation does not affect the DNA binding ability of HNF-1A

**Figure 4:**
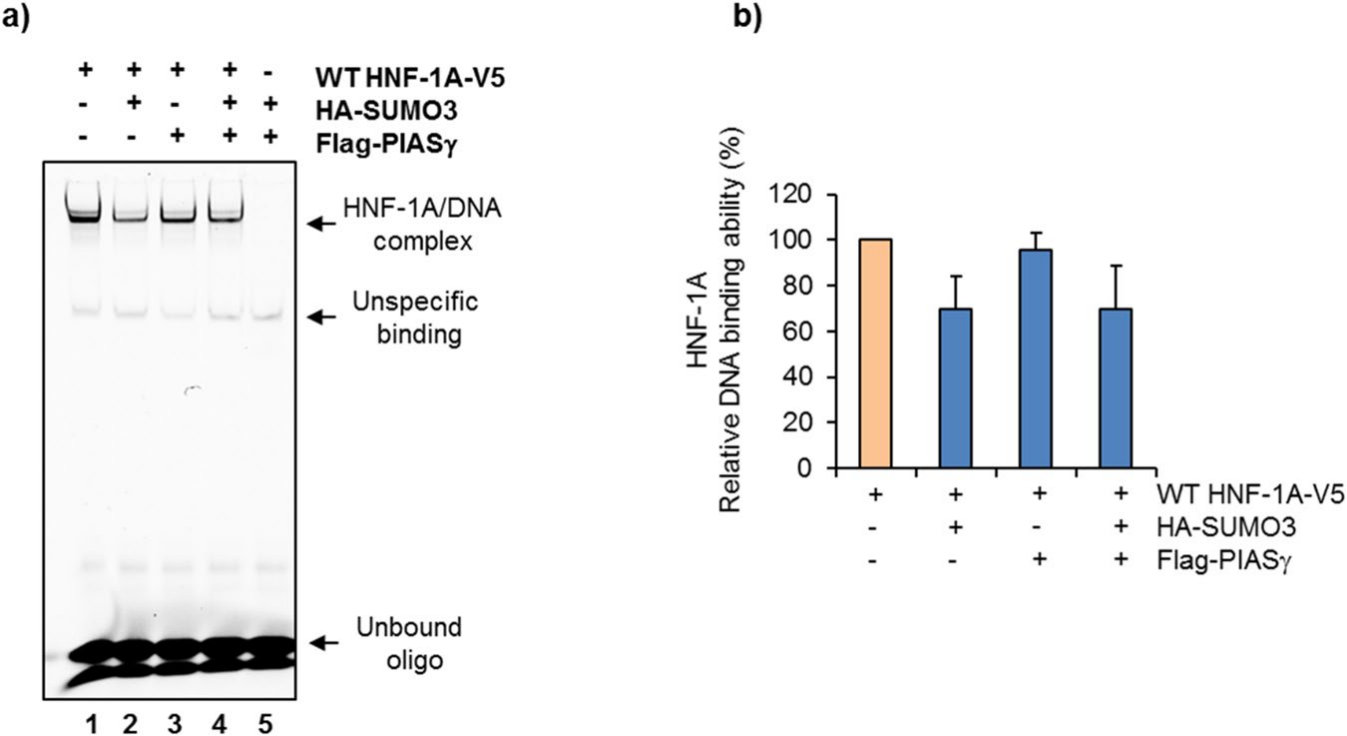
PIASγ does not influence the DNA binding ability of HNF-1A. (**a**) The DNA-binding ability of HNF-1A was assessed in nuclear fractions isolated from HeLa cells transiently transfected with WT HNF-1A or empty vector, and co-transfected with SUMO-3 and PIASγ. HNF-1A-DNA binding was analyzed by electrophoretic mobility shift assay (EMSA) after HNF-1A incubation with a Cy5 labeled DNA oligo (corresponding to HNF-1 binding site in the rat albumin). Bound complexes were separated on a 6% DNA retardation gel and the fluorescence signal was detected at 670 nm. Full-length gel is presented in Supplementary Fig. S6. (**b**) Quantification of binding by densiometric analysis is presented relative to WT alone (set to 100%). Each bar represents a mean of three independent experiments ±SD (n = 3).

Functional investigations indicate that the transcriptional activity of some transcription factors are repressed by SUMOylation, either by recruiting repressive co-factors^24^, by influencing their stability^25,26^ or by affecting their DNA-binding ability^27^. Furthermore, members of the PIAS family such as PIAS1 and PIAS3 have been reported to inhibit the DNA-binding activity of their protein targets^28,29^. We therefore aimed to investigate whether reduction in HNF-1A transactivation mediated by the SUMOylation machinery could be attributed to loss of DNA binding by HNF-1A. Thus, equal amounts of nuclear fractions isolated from HeLa cells overexpressed with WT HNF-1A, and in the presence of SUMO-3 and PIASγ, were incubated with a Cyanine 5 label probe, corresponding to the HNF-1 binding site of the rat albumin promoter and analyzed by Electrophoretic mobility shift assay (EMSA). Specificity of oligonucleotide binding was also verified by a competition assay using increasing amounts of unlabeled oligonucleotide (Supplementary Fig. S4). DNA-HNF-1A protein complexes were observed in all samples (except for negative control), however, with varying intensities indicating altered DNA-binding affinity (Fig. 4). More specifically, the presence of SUMO-3 alone or in combination with PIASγ seemed to only slightly impair the DNA binding ability of HNF-1A, indicated by reduced intensity of bands corresponding to the HNF-1A-DNA complexes (Fig. 4a). Upon quantification, these bands were estimated to represent a DNA binding ability of ∼70% compared to HNF-1A alone, set to 100% (Fig. 4b). On the contrary, co-expression with PIASγ alone did not reduce the DNA binding ability of HNF-1A, demonstrated by similar band intensity as HNF-1A expressed alone, and estimated to ∼95% (Fig. 4b).

### SUMOylation does not alter the total nuclear level of HNF-1A

**Figure 5:**
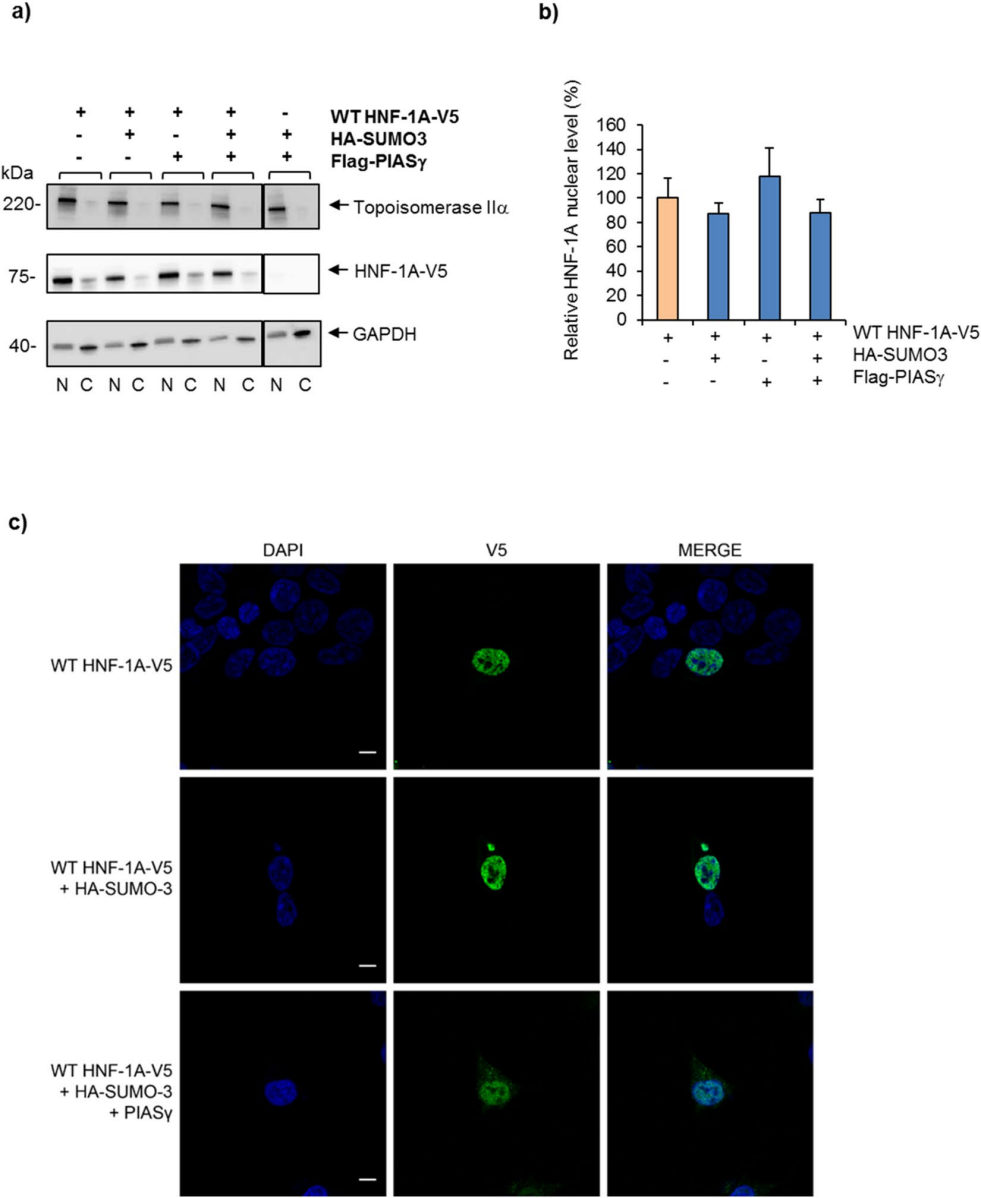
SUMOylation is not required for nuclear localization of HNF-1A. (**a**) Subcellular localization of HNF-1A was assessed in HeLa cells transiently transfected with V5-tagged WT HNF-1A together with constructs expressing HA-tagged SUMO-3 and/or Flag-tagged PIASγ (as indicated), and analyzed by cellular fractionation, SDS-PAGE and immunoblotting. ‘N’ indicates nuclear fractions, whereas ‘C’ indicates cytosolic fractions. The purity of the fractions was verified using antibodies against the nuclear- (topoisomerase IIα) and the cytosol-specific (GAPDH) marker proteins. Full-length blots are presented in Supplementary Fig. S7. (**b**) The level of HNF-1A in the nuclear fractions was normalized to topoisomerase IIα. The results are presented relative to the nuclear level of HNF-1A in cells transfected with HNF-1A alone. Each bar represents a mean of three independent experiments ±SD (n = 3). (**c**) HEK293 cells were transiently transfected with constructs expressing V5-tagged WT HNF-1A (in green) alone or in combination with HA-tagged SUMO-3 and/or Flag-tagged PIASγ. Localization of HNF-1A was visualized by immunofluorescence analysis using anti-V5 antibody and confocal microscopy. This experiment was replicated in two independent experimental days (n = 2). Cell nuclei were stained with DAPI (blue). Scalebar, 10 µm.

Previous studies have linked SUMOylation as effector of the nuclear localization of certain transcriptional regulators^30^, by showing for instance that SUMOylation negatively regulates their transcription by controlling their nuclear availability and subnuclear localization^31,32^. Based on this, we next aimed to investigate whether SUMOylation affects the nuclear level of HNF-1A. The presence of SUMO-3 and PIASγ did not have any notable effect on the nuclear fraction level of HNF-1A, indicating that the total nuclear level of HNF-1A is not affected by the SUMOylation machinery (Fig. 5a,b). The level of cytosolic HNF-1A, on the other hand, seemed slightly reduced in the presence of the SUMOylation machinery, from our immunoblots (Fig. 5a).

To further evaluate the effect of SUMOylation on the subcellular localization of HNF-1A we used immunofluorescence and confocal microscopy of transiently transfected HEK293 cells. These analyses revealed that HNF-1A alone was localized throughout the nucleus, apart from the nucleoli, in line with what was reported previously^33^. The same distribution was observed in the presence of the SUMOylation machinery (Fig. 5c), and confirming the findings in Fig. 5a,b. Confirmation of the slightly reduced cytosolic levels of HNF-1A was difficult by immunofluorescence, due to weak cytosolic staining compared to strong nuclear signals (Fig. 5c).

### PIASγ interacts with HNF-1A and reduces the protein level of HNF-1A

**Figure 6:**
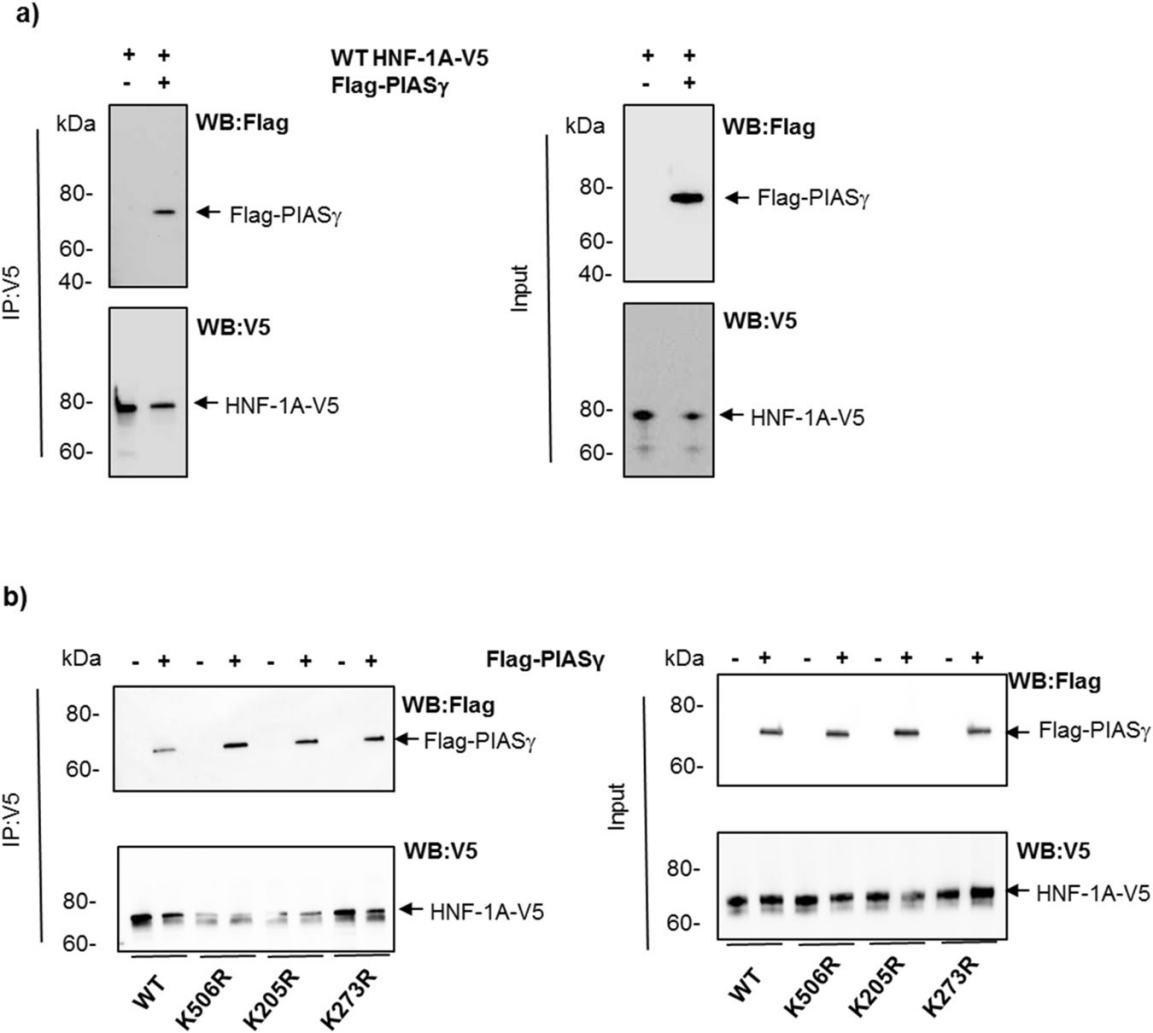
PIASγ interacts with HNF-1A. (**a**) Co-immunoprecipitation analysis of HNF-1A and PIASγ from HEK293 cells transiently transfected with V5-tagged HNF-1A or empty vector together with Flag-tagged PIASγ. Cells were lysed and subjected to immunoprecipitation using anti-V5 antibody. The precipitates (left) and 10 µg input (right) were separated by SDS-PAGE and immunoblotting using anti-Flag and anti-V5 antibodies. This experiment was replicated in two independent experimental days (n = 2). (**b**) Mutation of K205, K273 and K506 in HNF-1A does not affect the binding of PIASγ to HNF-1A. HEK293 cells were transiently transfected with WT HNF1A, K205R, K273R or K506R, together with Flag-tagged PIASγ. Lysates were analyzed similarly as described in (**a**) using anti-V5 antibody immunoprecipitation, SDS-PAGE analysis and immunoblotting. Full-length blots are presented in Supplementary Fig. S8. This experiment was replicated in two independent experimental days (n = 2).

**Figure 7:**
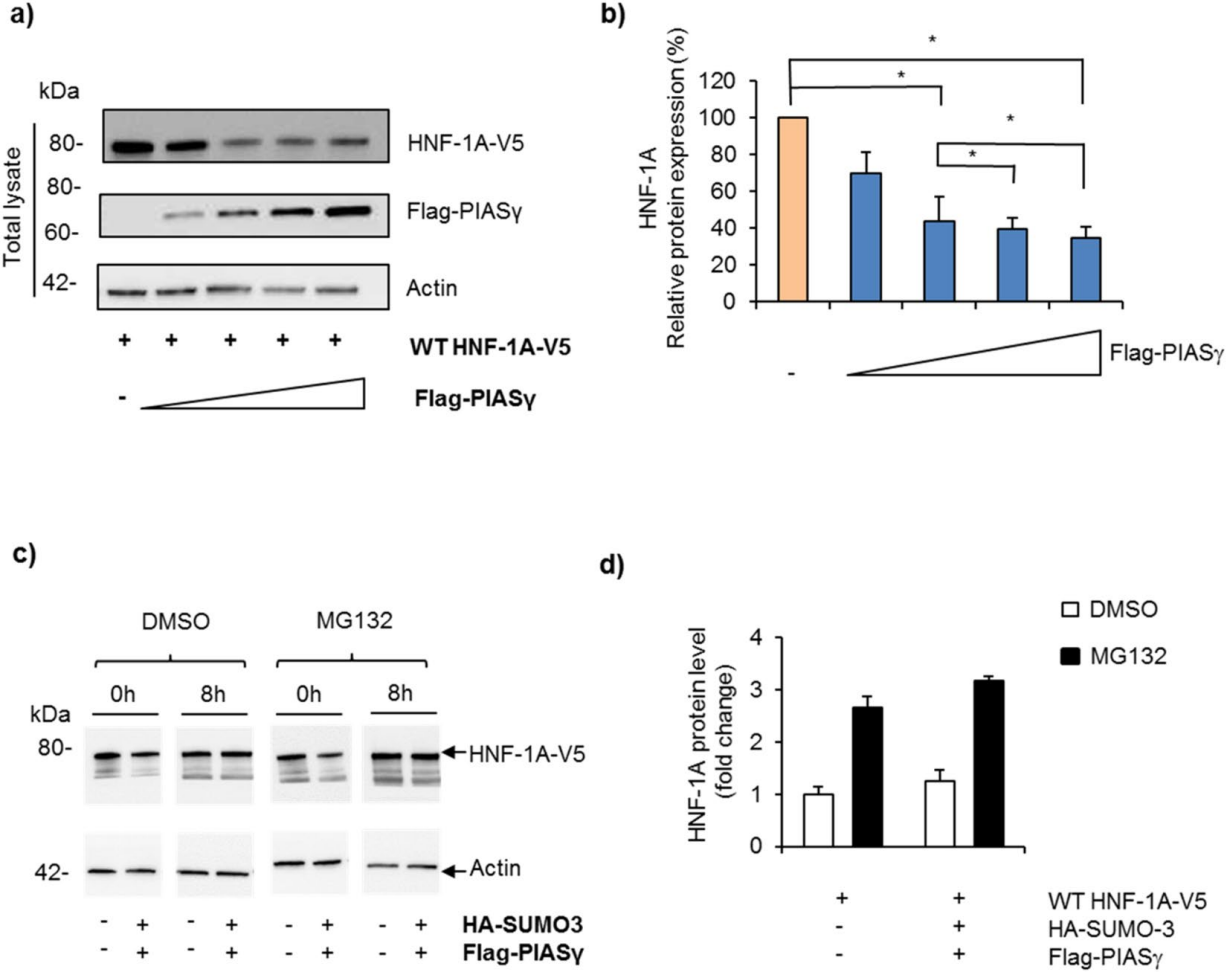
PIASγ reduces the cytosolic HNF-1A protein level in a dose- dependent manner. (**a**) Effect of PIASγ on HNF-1A protein in cleared lysates from transiently transfected HEK293 cells, with V5-tagged HNF-1A together with increasing amounts of Flag-tagged PIASγ (0.5–2 µg), and analyzed by SDS-PAGE and immunoblotting using indicated antibodies. Full-length blots are presented in Supplementary Fig. S9. (**b**) The level of HNF-1A was normalized to actin and presented relative to the level of HNF-1A alone. The results are shown as mean of four independent experiments ±SD (n = 4). *Indicates p < 0.05. (**c**) HEK293 cells were transiently transfected with V5-tagged HNF-1A in the presence or absence of HA-SUMO-3 and Flag-tagged PIASγ. Post-transfection, cells were treated with 10 µg MG132 or DMSO for 8 hours, and protein from cleared lysates was analyzed by SDS-PAGE and immunoblotting using indicated antibodies. Full-length blots are presented in Supplementary Fig. S10. (**d**) Quantification of proteins shown in c) by densiometric analysis. The level of HNF-1A was normalized to the loading control (actin). Each column represents the mean fold difference in the MG132-treated samples versus DMSO-treated control on two different experimental days ± SD (n = 2).

Next, we examined whether the reduction in HNF-1A activity by PIASγ could be mediated by an interaction between HNF-1A and PIASγ. HEK293 cells were firstly overexpressed with HNF-1A and PIASγ and co-immunoprecipitation experiments were performed (Fig. 6). In the HNF-1A immunoprecipitated samples analyzed by Flag-antibody, a PIASγ-specific band at ∼75 kDa was observed, only present in the sample transfected both with WT HNF-1A and PIASγ, and not in the control sample representing HNF-1A alone (Fig. 6a), indicating that PIASγ interacts with HNF-1A. We further overexpressed the K205R, K273R and K506R mutants (Fig. 6b) and the triple mutant K205RK273RK506R (Supplementary Fig. S5) in the presence or absence of PIASγ and performed a similar co-immunoprecipitation experiment. Similarly to WT, all mutants were capable of binding to PIASγ (Fig. 6b and Supplementary Fig. S5), demonstrated by a PIASγ specific band in each sample, and indicating that the binding of PIASγ to HNF-1A is independent of any of these SUMO-target lysine residues. Interestingly, we also noticed that overexpression of PIASγ resulted in slightly reduced intensity of the band representing unmodified HNF-1A band (Fig. 6a). This effect of PIASγ on HNF-1A protein level was also observed in our initial immunoprecipitation experiments (Fig. 2b). Therefore, to assess whether PIASγ affects the stability of HNF-1A, HNF-1A was overexpressed in the presence of increasing amounts of PIASγ plasmid in HEK293 cells. A dose-dependent increase in PIASγ plasmid significantly reduced the HNF-1A protein level detected in the supernatant fraction after RIPA lysis (Fig. 7a,b). Altogether, these results indicate that PIASγ interacts with HNF-1A either directly or indirectly through other unknown modifying factors, and that this leads to a reduction in the total HNF-1A protein level.

To explore whether the PIASγ mediated reduction in cytosolic HNF-1A protein could be explained by increased proteasomal degradation of HNF-1A, as shown for other proteins^34^, HEK293 cells were overexpressed with HNF-1A in the presence/absence of the SUMOylation machinery, and subsequently treated with the proteasomal inhibitor MG132 (Fig. 7c,d). An increase in HNF-1A protein from cleared lysate after MG132 treatment and RIPA lysis was detected, confirming that HNF-1A is regulated by the proteasomal degradation system in the presence or absence of SUMO-3 and PIASγ, as shown before^35^. No difference, however, in the total level of HNF-1A protein was detected after inhibition of the proteasomal degradation system, indicating that the PIASγ mediated reduction of HNF-1A protein level observed in Fig. 7a,b is not executed by the proteasomal system.

### PIASγ targets HNF-1A to the nuclear periphery and leads to repression of known HNF-1A regulated target genes

**Figure 8:**
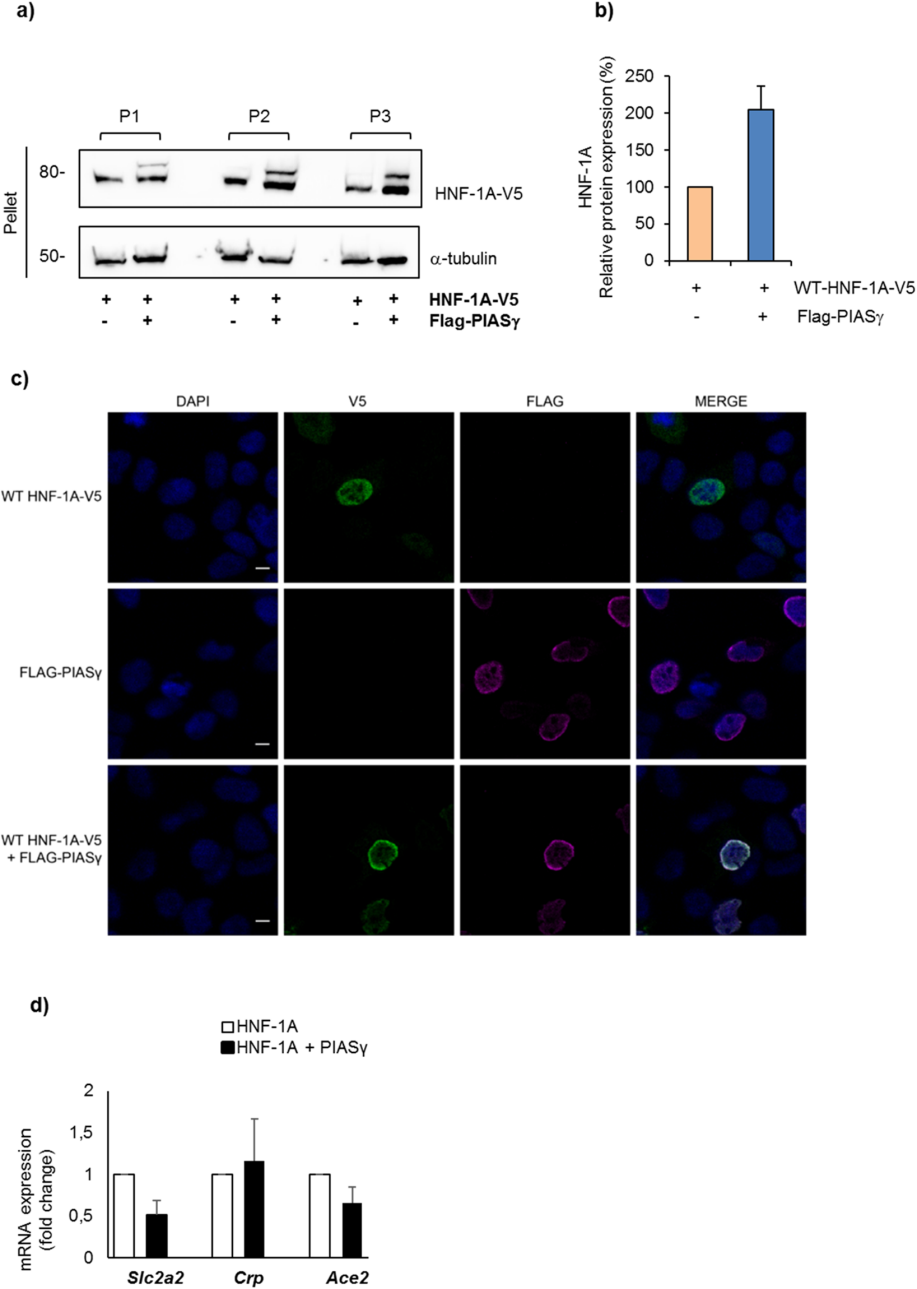
PIASγ translocates HNF-1A to the nuclear periphery and leads to reduced expression of known HNF-1A target genes. (**a**,**b**) After RIPA lysis, pelleted fractions from HEK293 cells transiently transfected with V5-tagged HNF-1A in the absence/presence of PIASγ were analyzed by SDS-PAGE and immunoblotting using indicated antibodies. The level of HNF-1A was quantified by densiometric analysis using the Image Lab v.6.0.0 software (BIO-RAD) and normalized to α-tubulin. Full-length blots are presented in Supplementary Fig. S11. Quantifications are presented relative to the level of HNF-1A alone. The results are shown as mean of three technical experiments (n = 1) ±SD. P indicates parallel. (**c**) HEK293 cells were transiently transfected with constructs expressing V5-tagged HNF-1A and/or Flag-PIASγ alone or in combination as indicated in the figure. HNF-1A (green) and PIASγ (magenta) were visualized by immunostaining for V5- and Flag-tag, respectively, and analyzed by confocal microscopy. Cell nuclei were stained with DAPI (blue). Co-localization of transfected HNF-1A and PIASγ is shown in the lower panel. Scalebar, 10 µm. (**d**) The mRNA expression of three known *HNF1A* targets genes (*Ace2*, *Slc2a2* and *Crp*) was investigated in MIN6 cells overexpressed with *HNF1A* in the absence or presence of *PIASy* and was determined by qPCR. Target gene expression was normalized to reference gene *Gapdh* and represented as mean fold change relative to HNF1A alone ±SD of two biological replicates (n = 2).

To further pursue the reason for the reduction in protein level of HNF-1A, in cleared RIPA lysates when PIASγ was present, we next investigated the pelleted fraction after RIPA lysis, containing cell debris, insoluble proteins and membrane fractions. Interestingly, we noticed a markedly increase in HNF-1A protein in the pelleted samples when PIASγ was present, relative to that in pellet fractions without PIASγ (Fig. 8a,b). These results suggest that PIASγ stimulates a subcellular translocation of HNF-1A to a membrane compartment where HNF-1A is not released by regular RIPA lysis.

We further investigated this effect of PIASγ on HNF-1A nuclear distribution by immunofluorescence experiments and confocal microscopy in transiently transfected HEK293 cells. Interestingly, co-expression of HNF-1A with PIASγ resulted in a redistribution of HNF-1A to the nuclear periphery, where it was observed to co-localize with PIASγ based on overlapping immunofluorescence signals (Fig. 8c). Therefore, this observation indicates that PIASγ sequesters HNF-1A to the nuclear periphery. To further explore if the HNF-1A/PIASγ interaction and re-localization of HNF-1A to the nuclear membrane would lead to a repression of HNF-1A regulated target genes, we performed qPCR of three known HNF-1A targets in MIN6 β-cells, i.e. *Ace2*^36^, *Slc2a2*^3^ and *Crp*^37^, and comparing mRNA levels in cells overexpressed with either *HNF1A* alone or *HNF1A* and *PIASγ* (Fig. 8d). Interestingly, the expression of *Ace2* and *Slc2a2* was reduced in the presence of PIASγ, while the level of *Crp* was unchanged.

## Discussion

Although mutations in *HNF1A* is the most prevalent inherited type of diabetes, the precise mechanism of how its gene product, HNF-1A is regulated, is largely unknown. Recent reports highlight the importance of balance of PTMs in insulin-producing β-cells for maintenance of glucose homeostasis, and in particular the role of SUMOylation^38,39^. In this study, we therefore investigated whether SUMOylation might be a crucial mechanism for the normal regulation of HNF-1A.

Different SUMO isoforms serve distinct functional roles by their modification of individual proteins^40^. For instance, while RanGAP1 is predominantly modified by SUMO-1, and to lesser extent by SUMO-3^41^, SUMO-3 predominantly modifies nuclear actin^42^. Our data supports that HNF-1A is preferentially modified by SUMO-3 compared to SUMO-1 (Fig. 1a). SUMO-3 also seemed to be more frequently conjugated to proteins, in general, in the HEK293 cells analyzed, consistent with previous reports showing that SUMO-3 has a substantially higher potential to modify cellular proteins than SUMO-1^41^.

Furthermore, the SUMO E3 ligase, PIASγ, increased the level of SUMOylated HNF-1A, which is in line with previous findings on the role of PIASγ as a catalyst of SUMOylation^43–45^.

SUMOylated HNF-1A appeared as multiple high molecular mass bands (Fig. 1). Since SUMO-3 is known to be conjugated both alone, or as SUMO chains, at the same target residue^46^, it is therefore possible that HNF-1A is modified by polySUMOylation by SUMO-3 at one target lysine site and/or sequential SUMOylation of multiple sites. Analyses by both *in silico* prediction tools and of SUMOylation level assessment of candidate lysine mutants identified K205 and K273 as the most important SUMOylation site residues (Fig. 2b,c). The relevance of K506 was more difficult to determine, since the K506R mutant was shown to be very unstable in the presence and in the absence of the SUMOylation machinery (Fig. 2b, Supplementary Fig. S3 and unpublished data). Thus it is possible that the reduction in detectable SUMOylated forms of K506R could be due to a reduction in the protein level of K506R, and concealing whether K506 is a true SUMOylation site or not. Since both the double mutant (K205RK273R) and triple mutant (K205RK273RK506R) possessed some degree of SUMOylation (Supplementary Fig. S2), this suggests that either i) there are more SUMOylation sites in HNF-1A than K205 and K273 or ii) there is some redundancy in SUMO-site specificity in HNF-1A, as previously referred to by others on different SUMO targets^47^.

In terms of functional consequence we found that SUMO-3/PIASγ significantly reduced HNF-1A transactivation activity (Fig. 3a). This repression seemed not to be dependent on HNF-1A SUMOylation at single lysines K205 and K273, since the transcriptional activity of the SUMOylation–deficient mutants (K205R and K273R) were also inhibited by PIASγ/SUMO3. By contrast, for the double/triple mutants K205RK273R(K506R), we did not observe any reduction in transactivation of PIASγ/SUMO-3 or by PIASγ alone (Fig. 3b), indicating that simultaneous SUMOylation of these lysines are important for the inhibitory effect observed by PIASγ.

A fact supporting the importance of K205, K273, and K506 within the protein structure of HNF-1A is that they are highly conserved among different species and they are also conserved in the paralog HNF-1B. Residue K205 and K273 are located in the DNA binding domain, while K506 is located in the transactivation domain (Fig. 2a). Worth noting is that the baseline transcriptional activity of all the SUMO-site lysine → arginine mutants was significantly lower compared to WT HNF-1A activity (Supplementary Fig. S3). One possible explanation for their reduced activity might be that these lysines represent important residues for the normal structure and activity of HNF-1A. A previous report on K205 shows that this lysine residue is important for inter-domain interaction, proper protein folding/structure and stability of HNF-1A, and that loss of this lysine (mutation K205Q) leads to reduced protein level due to instability, and hence reduced detectable transcriptional activity^48^. Residue K506 seems also essential for HNF-1A stability and activity as the mutant K506R, in our hands, demonstrated the lowest level of transcriptional activity of the single mutants (Supplementary Fig. S3), and our immunofluorescence analyses (data not shown) also indicated reduced protein level by weak staining. As mentioned earlier, loss of function mutations in *HNF1A* cause the most common form of monogenic diabetes (HNF-1A-MODY;MODY3), a disorder characterized by reduced glucose-stimulated insulin secretion^5^. Interestingly, missense variants at K205 (K205Q) and at K273 (K273N) have been associated with MODY3^48–50^. This further supports the relevance of these lysine residues for normal HNF-1A function. Accordingly, the normal regulation by SUMOylation of these diabetes-associated variants, and SUMOylation site deficient mutants, is most likely impaired (Fig. 2b).

Another important aspect of SUMO-conjugation of a target protein is the reversible action by SENPs. Interestingly, SENP-1 has recently been shown to be activated by glucose^51^. Our study confirmed that SENP-1 successfully de-SUMOylated HNF-1A (Fig. 1b) and Hnf-1a (Supplementary Fig. S1). Given that SUMOylation by SUMO-3 has a repressive effect on HNF-1A transcriptional activity, it might be likely that conditions of high glucose will activate SENP-1, and subsequent lead to de-SUMOylation of HNF-1A. Ultimately, this may result in a more active HNF-1A protein under high glucose.

In our study, the E3 ligase PIASγ revealed to be a new interacting partner of HNF-1A (Fig. 6) and was shown to reduce HNF-1A transactivation significantly using a rat albumin promoter in a luciferase assay (Fig. 3a). Further, we showed that *HNF1A/PIASγ* co-transfection reduced the expression of known HNF-1A target genes, i.e. *Ace2* and *Slc2a2*, in MIN6 cells (Fig. 8d). Previous studies have shown that PIASγ can repress transcription either by recruiting transcriptional corepressors^52^, or by re-localization of the transcription factor within the nucleus^45^. A PIASγ mediated translocation of HNF-1A to the nuclear membranes is supported in our study, and shown by immunofluorescence and confocal imaging analyses, as well as analyses of cell pelleted fractions (Fig. 8). PIASγ has previously been reported to translocate the CCAAT/enhancer binding protein δ from transcriptionally active nuclear foci to the nuclear periphery, and as a result represses the transcriptional ability of protein δ^53^. According to previous studies, the nuclear periphery, and especially the inner nuclear envelope, are cellular areas that have been involved in the modulation of gene expression^54^. More specifically, inner nuclear membrane proteins such as lamins and emerins have shown to physically interact with several transcription factors when these enter the nucleus. By this, they restrict transcription factor access to their target genes in the nucleosome where transcription occurs, and hence induce transcriptional repression^55,56^. We believe that the repression by PIASγ on HNF-1A transactivation may be due to PIASγ-mediated sequestration of HNF-1A to the nuclear periphery and subsequent restricted access of HNF-1A to its target genes, as demonstrated by reduced mRNA expression for *Ace2* and *Slc2a2* observed in the presence of PIASγ (Fig. 8d). An attractive hypothesis is that this PIASγ-mediated repression of HNF-1A activity occurs at low glucose conditions and in β-cell resting state.

In summary, we report here that SUMOylation of HNF-1A represents a novel post translational regulatory mechanism of HNF-1A. Further, we have identified PIASγ as a novel HNF-1A interaction partner that enhances the SUMOylation of HNF-1A. Furthermore, the PIASγ/HNF-1A interaction leads to re-localization of HNF-1A to the nuclear periphery, leading to restricted access to its target genes, thereby reducing the overall HNF-1A transcriptional activity. This new knowledge is highly relevant for precision medicine in diabetes as a novel mechanism for HNF-1A regulation and altered function by PIASγ, which reveal potential new targets for drug development in HNF-1A associated diabetes.

## Methods

### Cell lines and transfection

MIN6 cells were grown in DMEM (4.5 g/L glucose) supplemented with 15% heat-inactivated FBS. HEK293 and HeLa cells were grown in DMEM (4.5 g/L glucose) including 10% heat-inactivated FBS. All growth media included penicillin/streptomycin. Transfections were performed using Lipofectamine-2000 (Invitrogen), if not stated otherwise.

### Constructs

*HNF1A* cDNA (NCBI Entrez Gene BC104910.1) in pcDNA3.1/V5-HisC was used (Life Technologies). All *HNF1A* Lys→Arg variants were constructed using the QuikChange XL Site-directed Mutagenesis Kit (Stratagene). Sequences of the primers are shown in Supplementary Table 1. Plasmids encoding human HA-tagged SUMO-1 and SUMO-3 (in pcDNA 3.1) were kindly provided by prof. Frauke Melchior (Heidelberg, Germany). Plasmids encoding human HA-tagged UBC9 (p3258 pCMV4) (#14438), Flag-tagged PIASγ (pCMV) (#15208) and Flag-tagged SENP1 (pCMV) (#17357) were from Addgene. The *Firefly* luciferase reporter vector pGL3-RA (Promega), containing the promoter of the rat albumin gene (nucleotide −170 to + 5) and the pRL-SV40 reporter vector (Promega) encoding the *Renilla* luciferase gene were used in the transactivation assay^57^.

### Luciferase assay

MIN6 cells were transiently co-transfected with plasmids expressing WT or variant *HNF1A* cDNA and HA-SUMO3, Flag-PIASγ and reporter plasmids expressing *Firefly* and *Renilla* luciferase. Post transfection, transactivation was measured using the Dual-Luciferase Assay System (Promega) in a Chameleon luminometer (Hidex). Luciferase activity was normalized by *Renilla* expression.

### Co-Immunoprecipitation

HEK293 cells transiently transfected with indicated plasmids and harvested using ice-cold IP Lysis/Wash Buffer (Thermo-Fisher) including 20 mM N-ethylmaleimide and protease inhibitor cocktail. Protein concentrations were determined in a Direct Detect Spectrometer (Merck Millipore). Co-immunoprecipitation (Co-IP) was performed using a V5-antibody (Thermo-Fisher; #R960-25) and the Pierce Co-IP kit (Thermo-Fisher). Samples were analyzed by SDS-PAGE and immunoblotting with mouse anti-V5 (Thermo-Fisher), rabbit anti-HA (Cell Signaling; #3724), rabbit anti-Flag (Cell Signaling; #2368) and horseradish peroxidase (HRP)-linked secondary antibodies.

### Endogenously SUMOylated HNF-1A

MIN6 cells, endogenously expressing Hnf-1a, were transiently transfected with the indicated plasmids and harvested in ice-cold BlastR lysis buffer supplemented with NEM and protease inhibitors (Cytoskeleton). The endogenously SUMOylated Hnf-1a proteins were isolated by immunoprecipitation using the Signal-Seeker SUMOylation 2/3 Detection Kit (Cytoskeleton), according to the manufacturer’s protocol. The eluted samples were analyzed by SDS-PAGE and immunoblotting using rabbit anti-HNF-1A (Cell Signaling; # 89670 S), rabbit anti-Flag (Cell Signaling; #2368) and mouse HRP-linked anti-SUMO2/3 (Cytoskeleton).

### Protein expression analysis

HEK293 cells were transiently co-transfected with plasmids expressing *HNF1A* cDNA and increasing amounts of Flag-PIASγ. Post transfection, cells were harvested in RIPA buffer (Thermo-Fisher) including 20 mM NEM and protease inhibitor cocktail. Protein concentrations were assessed by Pierce™ BCA Protein Assay kit (Thermo-Fisher). Pelleted samples were further treated with Benzonase (Sigma-Aldrich) for 30 min 37 °C. Samples were analyzed by SDS-PAGE and immunoblotting using mouse anti-V5 (Thermo-Fisher), rabbit anti-Flag (Cell Signaling), goat anti-Actin (Santa Cruz; sc-1615) and anti-alpha Tubulin-HRP (Abcam; Ab40742) antibodies.

### Electrophoretic mobility shift assay (EMSA)

A cyanine 5 labelled oligonucleotide (Sigma Aldrich) of PE56 double stranded DNA fragment (5′-TGTGGTTAATGATCTACAGTTA-3′) containing the rat albumin sequence (−63/−41) was used. The DNA binding reaction was performed using 10 µg nuclear fractions from transiently transfected HeLa cells and the Odyssey EMSA buffer kit (LI-COR Biosciences).

### Cell fractionation

HeLa cells transiently transfected with indicated plasmids were harvested in PBS post transfection. The cytosolic fraction was collected by re-suspending the cell pellet in buffer A (10 mM Hepes pH 7.8, 1.5 mM MgCl_2_, 10 mM KCl, 0.1% IGEPAL, 0.5 mM DTT, EDTA-free protease inhibitor cocktail) followed by incubation for 30 min on ice and centrifugation at 17 900 × g for 5 min at 4 °C. Next, the nuclear pellet was washed twice with buffer A and further re-suspended in buffer B (20 mM Hepes pH 7.8, 420 mM NaCl, 1.5 mM MgCl_2,_ 0.2 mM EDTA, 0.5 mM DTT, EDTA-free protease inhibitor cocktail) followed by incubation on ice for 30 min vortexing every minute. The nuclear fraction was further collected by centrifugation at 17 900 × g for 15 min 4 °C. The protein concentration was determined using the Pierce™ BCA Protein Assay kit (Thermo-Fisher). Each fraction was subjected to SDS-PAGE and immunoblotting using rabbit anti-HNF1A (Cell Signaling; #89670) and the purity of fractions was verified using antibodies against the nuclear- and cytosol- specific marker proteins topoisomerase IIα (Cell Signaling; #12286) and GAPDH (Santa Cruz; sc-47724), respectively.

### Immunofluorescence and Confocal microscopy

HEK293 cells were plated on to coverslips and transiently transfected using JetPrime transfection reagent (PolyPlus). Post transfection, cells were fixed using 3% paraformaldehyde for 30 min and permeabilized in 1x PBS, 0.1% Tween 20 (PBS-T), 0.1% Triton X-100 for 20 min at RT and further incubated in 5% goat serum diluted in PBS-T for 30 min at RT. Cells were then incubated with mouse anti-V5 (1:500; Thermo-Fisher) and/or rabbit anti-Flag tag (1:200; Thermo-Fisher; PA1-984B). The secondary antibodies anti-mouse IgG-Alexa Flour 488 (Thermo-Fisher; A11017) and/or anti-rabbit IgG-Alexa Flour 594 (Thermo-Fisher; A11037) were used at 1:200. Cells were washed in PBS-T overnight at 4 °C and coverslips embedded in ProLong Gold with DAPI (Thermo-Fisher) prior to imaging using TCS SP5 AOBS confocal microscope (Leica Microsystems).

### RNA isolation and gene expression analysis by quantitative real-time polymerase chain reaction (qPCR)

Total RNA was isolated using the RNeasy mini kit (QIAGEN) from MIN6 cells, transiently transfected with plasmids expressing human *HNF1A* cDNA and/or human *PIASγ*. During RNA isolation, an including on-column DNase digestion step using the RNase-Free DNase set (QIAGEN) was also included to eliminate genomic DNA contamination. cDNA was next synthesized from the total RNA (500 ng) of each sample using the SuperScript VILO cDNA Synthesis Kit (Thermo Fisher) and further diluted five times in RNase free water. For the qPCR analysis, the TaqMan Universal PCR master mix (Applied Biosystems) and the following TaqMan FAM probed (Applied Biosystems) were used: human HNF1A (Hs00167041_m1), human PIASγ (Hs00249203_m1), mouse Ace2 (Mm01159003_m1), mouse Crp (Mm00432680_g1), mouse Slc2a2 (Mm00446229_m1) and mouse Gapdh (Mm99999915_g1). The amplification reaction was carried out in the ABI Prism 7500 (Thermo-Fisher).Each reaction was carried out in three replicates and the comparative CT method was used for the relative quantification of the amount of mRNA in each sample normalized to the gapdh transcript levels.

### Statistical analysis

All data are presented as mean ± standard deviation (STD) and experiments were performed at least on three independent occasions unless otherwise specified. Statistical analysis was performed using a two- tailored Student’s t-test and a p value < 0.05 was considered statistically significant.

## Electronic supplementary material

Supplementary information
