# Supplementary material for: The E3 SUMO ligase PIASγ is a novel interaction partner regulating the activity of diabetes associated hepatocyte nuclear factor-1α
Source: Sci Rep. 2018 Aug 24;8:12780. doi: 10.1038/s41598-018-29448-w (PMC6109179; doi:10.1038/s41598-018-29448-w)

The E3 SUMO ligase PIASγ is a novel interaction partner regulating the activity of diabetes associated hepatocyte nuclear factor-1α

Alba Kaci, Magdalena Keindl, Marie H. Solheim, Pål R. Njølstad, Lise Bjørkhaug and Ingvild Aukrust

Supplementary Information

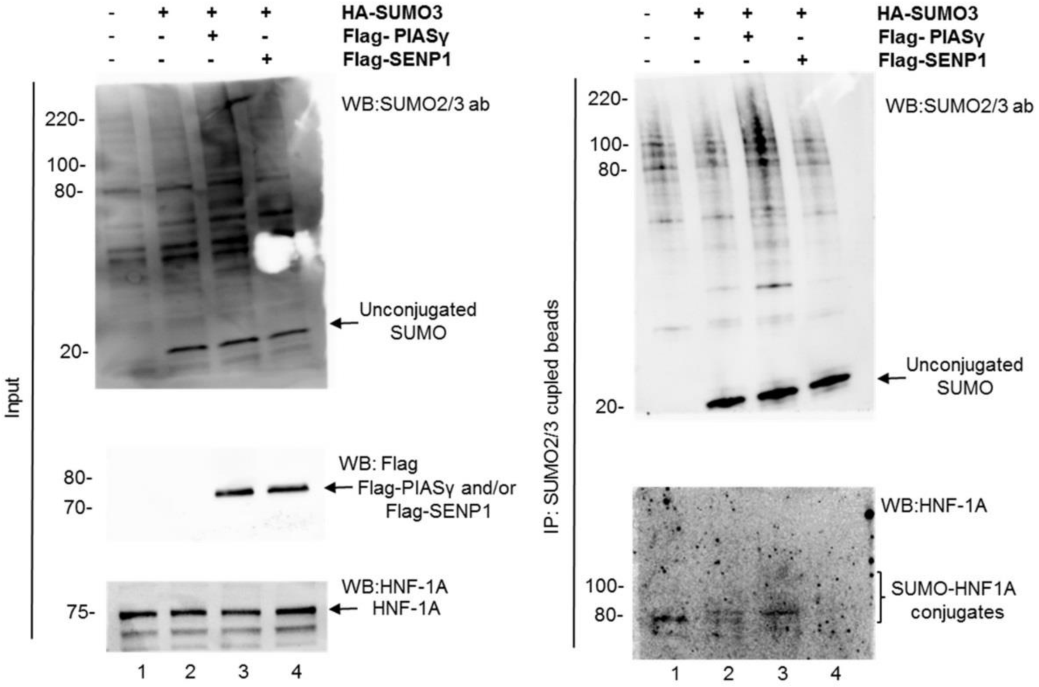

Figure S1. Endogenous mouse Hnf-1a in MIN6 cells is also a target of SUMO-3 modification. SUMOylation of endogenous mouse Hnf-1a analyzed in MIN6 cells transiently transfected with HA-tagged SUMO-3, and/or Flag-tagged PIASγ and Flag-tagged SENP1. Cells were lysed in the presence of N-Ethylmaleimide and protease inhibitors, and subjected to immunoprecipitation using anti-SUMO2/3 coupled beads. The precipitate (right panel) and input (left panel) samples were separated by SDS-PAGE and analyzed by immunoblotting using anti-SUMO2/3, anti-HNF-1A and anti-Flag antibodies. This experiment was performed once (n=1).

a)

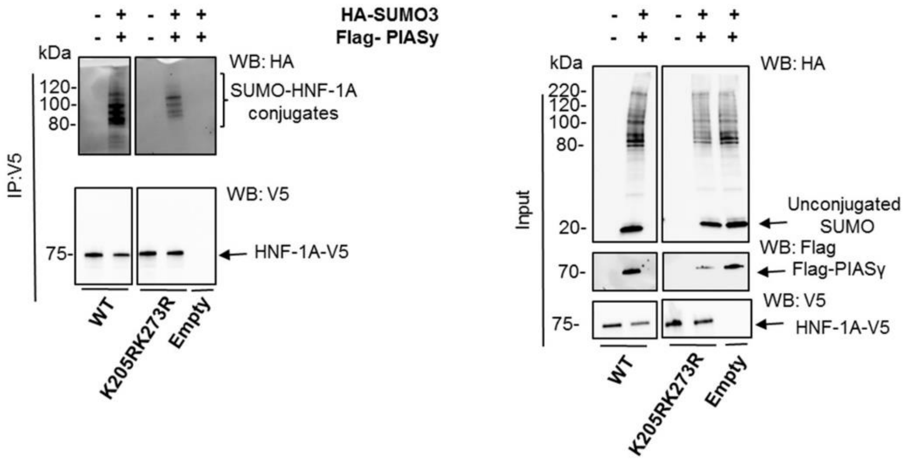

b)

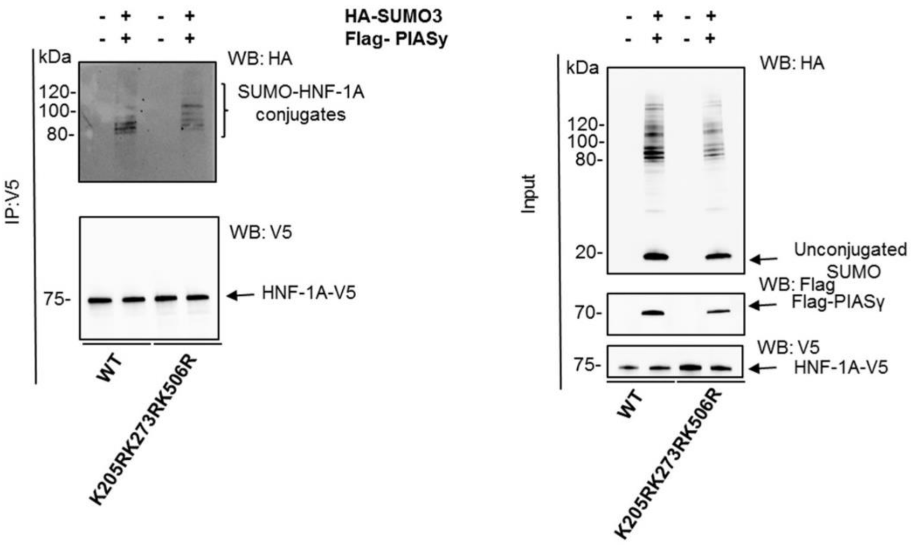

Figure S2. Combines loss of residues K205, K273 and/or K506 resulted in reduced SUMOylation upon lysine (K) to arginine (R) substitution. (a and b) Cells were transfected with V5-tagged HNF-1A (WT or mutants) together with HA-tagged SUMO-3 and Flag-tagged PIASγ. Lysates were collected in the presence of NEM and protease inhibitors and subjected to immunoprecipitation using anti-V5 antibody. The precipitates were separated by SDS-PAGE and analyzed by immunoblotting using anti-HA and anti-V5 antibodies. This experiment was performed once (n=1).

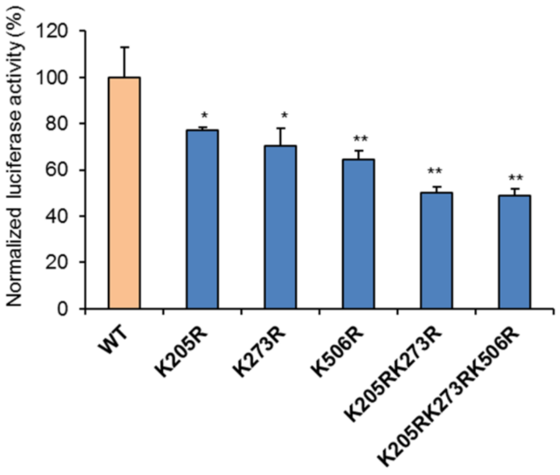

Figure S3. Loss of K205, K273 and K506 (to arginine) modulate negatively the activity of HNF-1A. MIN6 cells were transiently transfected with V5-tagged WT, single mutants (K205R, K273R, K506R), double mutant (K205RK273R) and triple mutant (K205RK273RK506R) individually, and co-transfected with reporter plasmids pGL3-RA and pRLSV40. Firefly luciferase activity was measured in cell lysates and normalized to Renilla luficerase expression. Each bar represents the mean nine readings ±SD; three parallel readings were conducted on each of three experimental days (n = 3). Measurements are given in fold activity relative to WT. * Indicates $p < 0.05$, ** indicates $p < 0.001$.

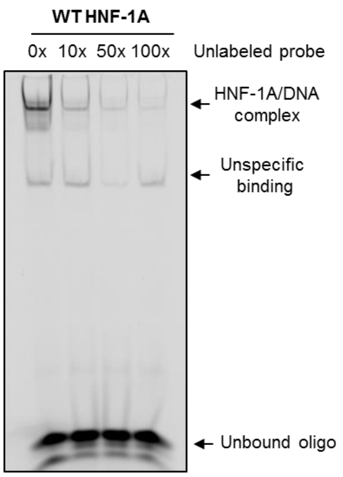

Figure S4. EMSA competition assay with unlabeled oligo. Nuclear fractions obtained from Hela cells transiently transfected with WT HNF-1A were incubated with increasing amounts of unlabeled DNA oligo corresponding to the HNF-1 binding site in the rat albumin promoter. Bound complexes were analyzed by EMSA, on a 6% DNA retardation gel and the fluorescence signal was detected at 670 nm.

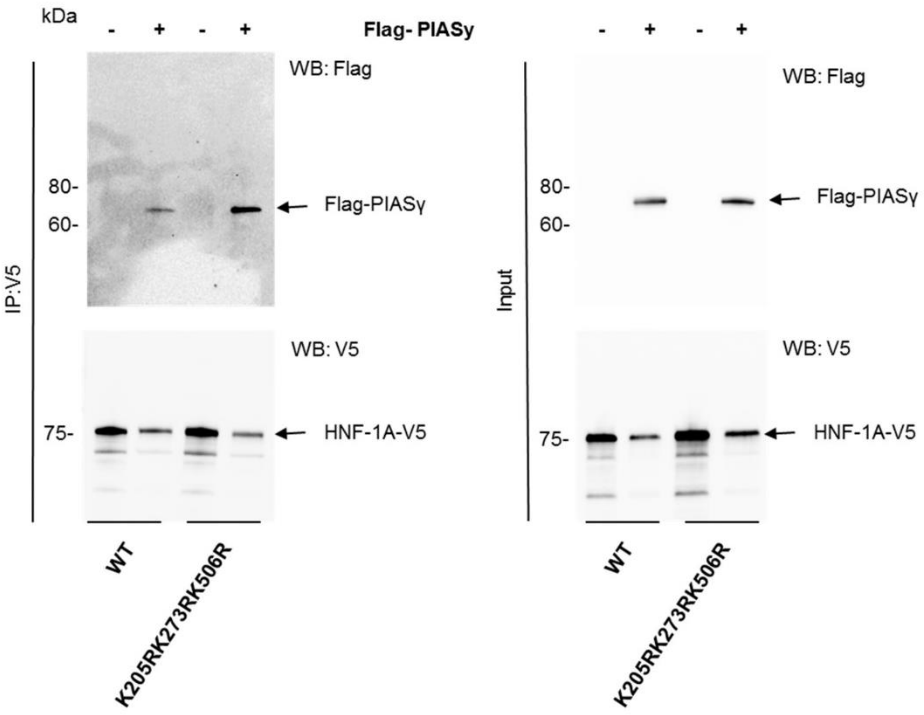

Figure S5. The trippel mutant K205RK273RK506R does not affect the binding of PIASγ to HNF-1A. HEK293 cells were transiently transfected with WT HNF1A, mutant K205RK273RK506R, together with Flag-tagged PIASγ. Cells were lysed and subjected to immunoprecipitation using anti-V5 antibody. The precipitates (left) and 10 µg input (right) were separated by SDS-PAGE and analyzed by immunoblotting using anti-Flag and anti-V5 antibodies. This experiment was performed once (n=1).

Table S1. Site directed mutagenesis primer sequences.

Primers	Sequence
K46R_forward	5’-GAAGGCCCCCTGGACAGGGGGGAGTCCTGCGGC-3’
K46R_reverse	5’-GCCGCAGGACTCCCCCCTGTCCAGGGGGCCTTC-3’
K158R_forward	5'-TCCCATGAAGACGCAGAGGCGGGCCGC-3'
K158R_reverse	5'-GCGGCCCGCCTCTGCGTCTTCATGGGA-3'
K205R_forward	5'-CGGAGGAACCGTTTCAGGTGGGGCCCAG-3'
K205R_reverse	5'-CTGGGCCCCACCTGAAACGGTTCCTCCG-3'
K222R_forward	5'-GCCTATGAGAGGCAGAGGAACCCTAGCAAGGAG-3'
K222R_reverse	5'-CTCCTTGCTAGGGTTCCTCTGCCTCTCATAGGC-3'
K273R_forward	5'-GTTTGCCAACCGGCGCAGAGAAGAAGCCTTCC-3'
K273R_reverse	5'-GGAAGGCTTCTTCTCTGCGCCGGTTGGCAAAC-3'
K506R_forward	5’- CTCTACAGCCACAGGCCCGAGGTGGCC-3’
K506R_reverse	5’-GGCCACCTCGGGCCTGTGGCTGTAGAG-3’

Figure S3: Full scans of blots from main text Figure 1a.

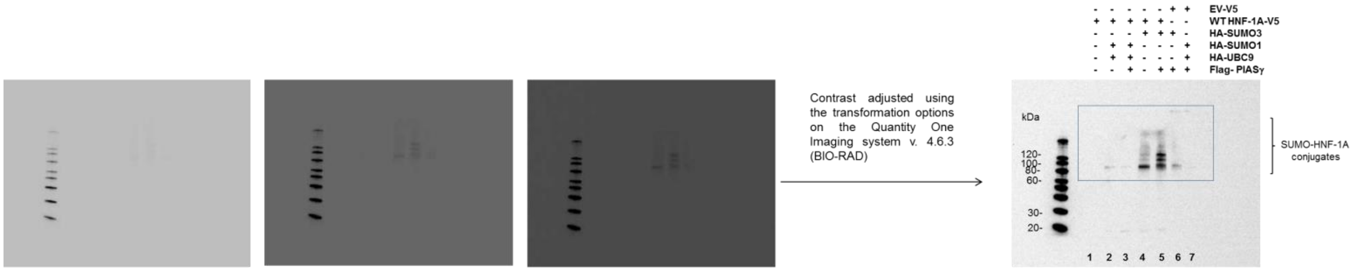

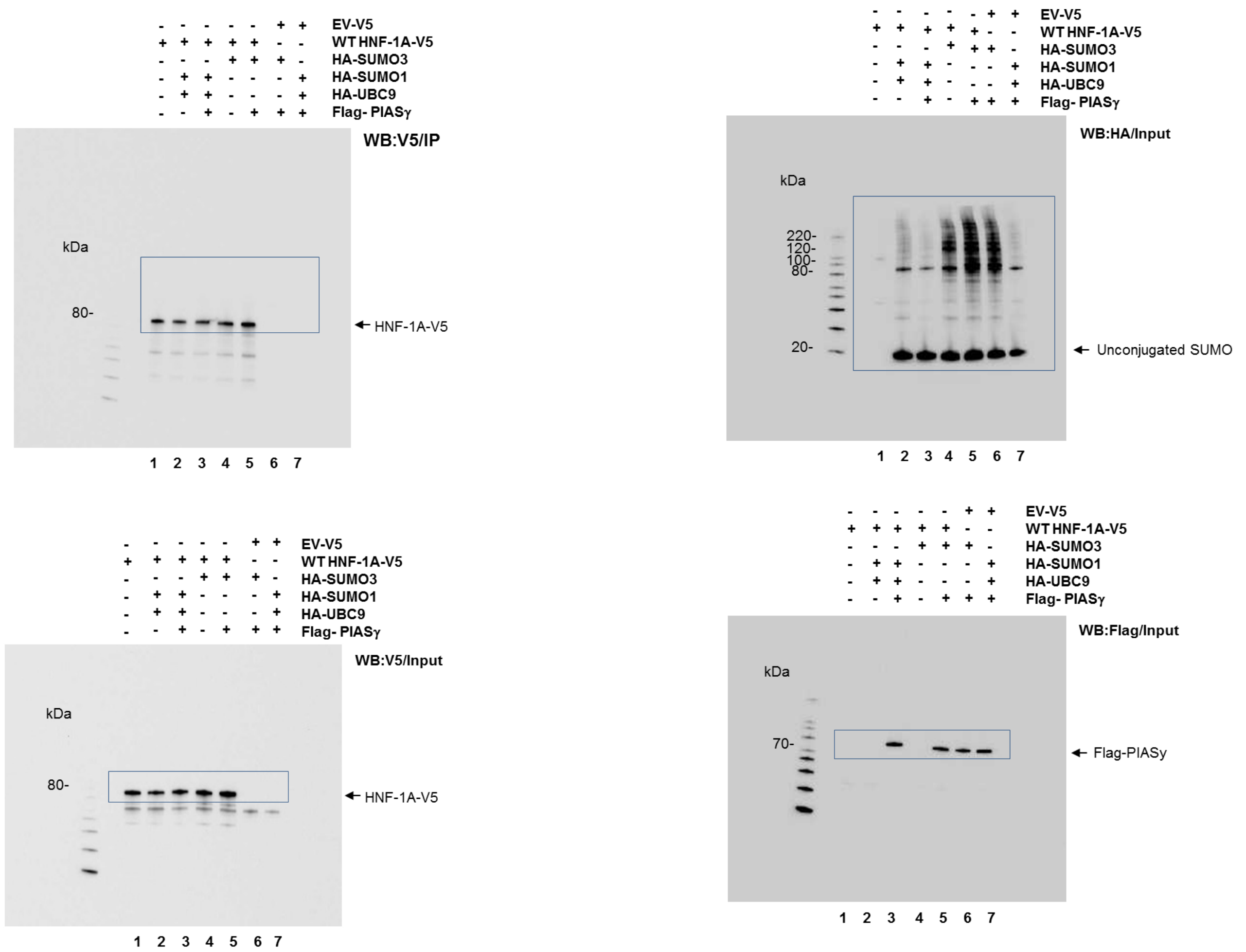

Figure S4: Full scans of blots from main text Figure 1b.

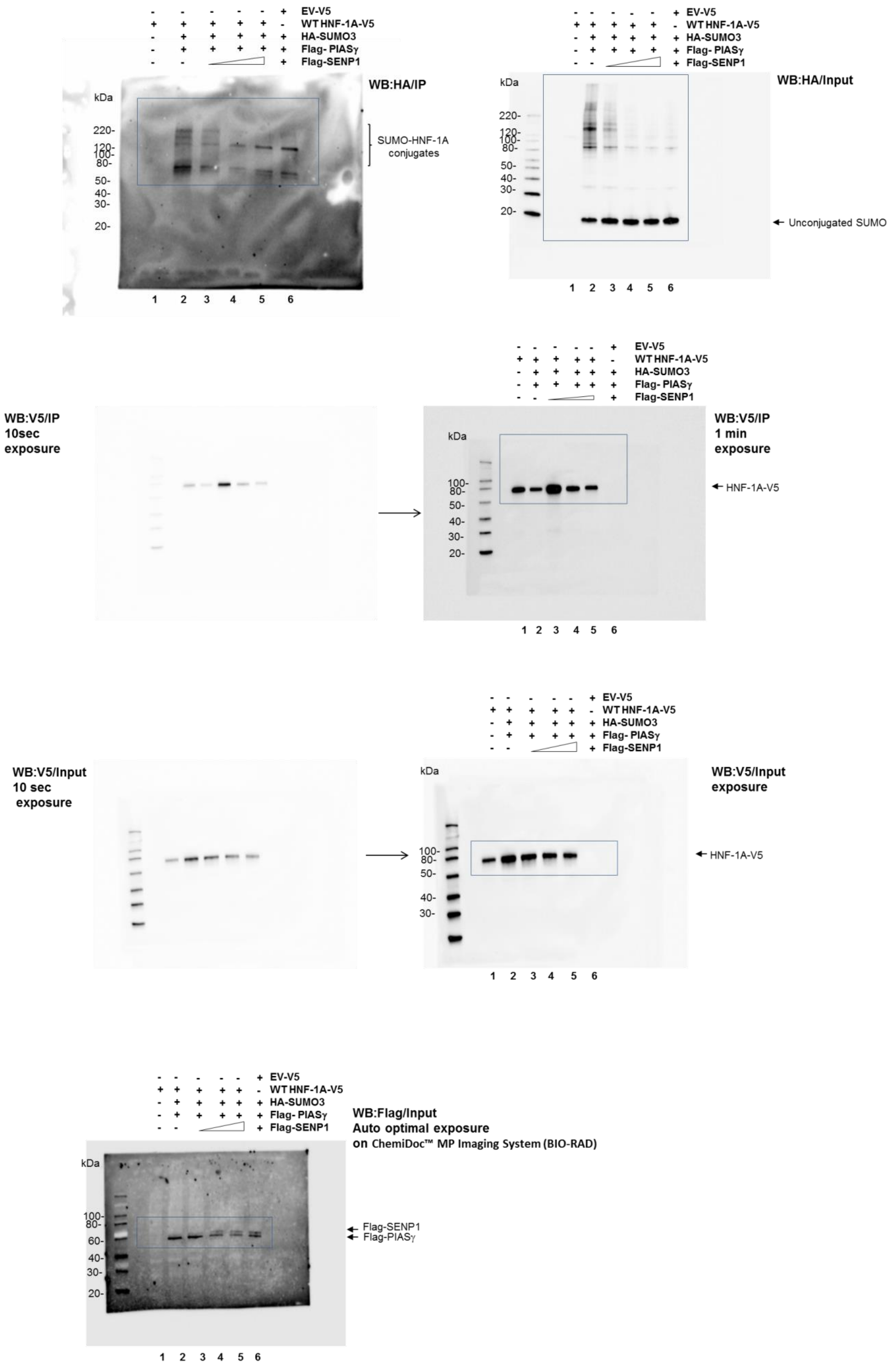

Figure S5: Full scans of blots from main text Figure 2b.

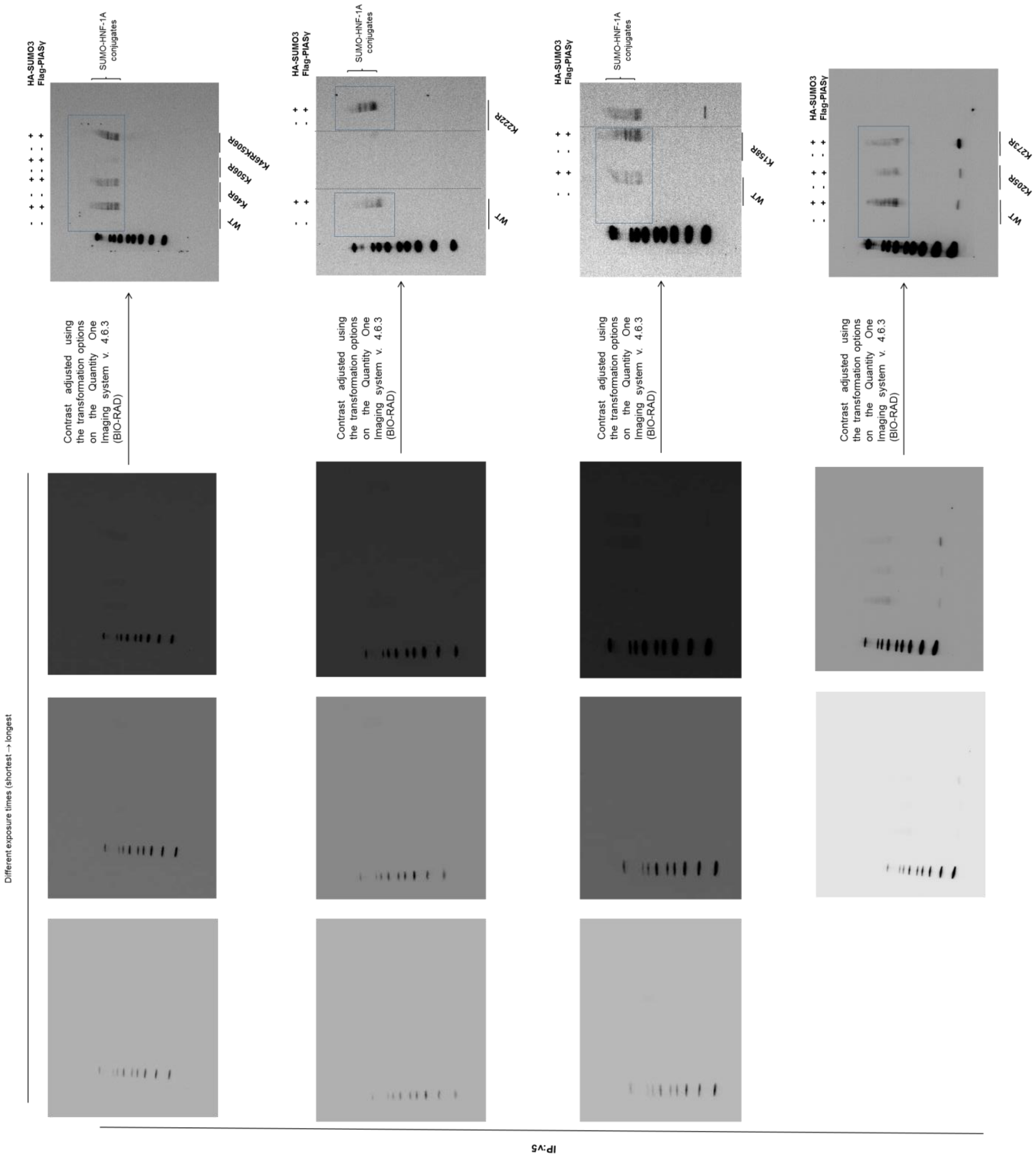

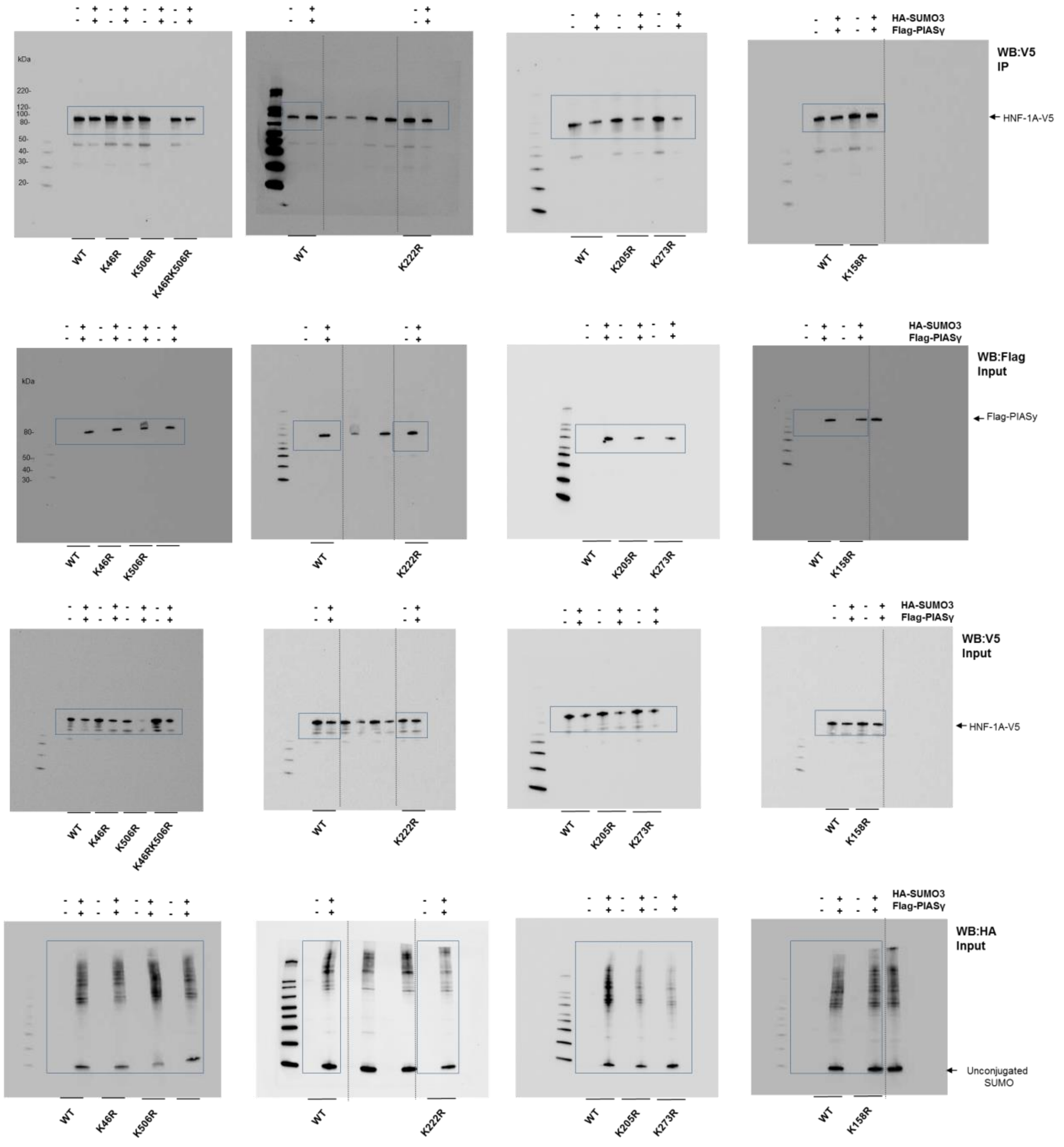
HA-SUMO3
Flag-PIASγ
WB:V5
IP
HNF-1A-V5
WB:Flag
Input
Flag-PIASγ
WB:V5
Input
HNF-1A-V5
WB:HA
Input
Unconjugated
SUMO
kDa
220-
120-
100-
80-
50-
40-
30-
20-
WT
K46R
K506R
K46RK506R
K222R
K205R
K273R
K158R

Figure S6: Full scans of blots from main text Figure 4a.

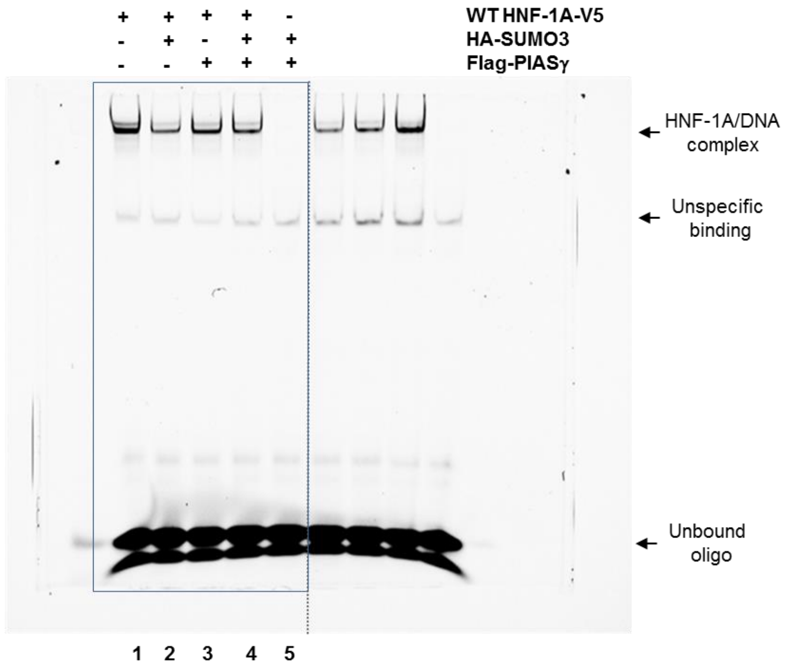

Figure S7: Full scans of blots from main text Figure 5a.

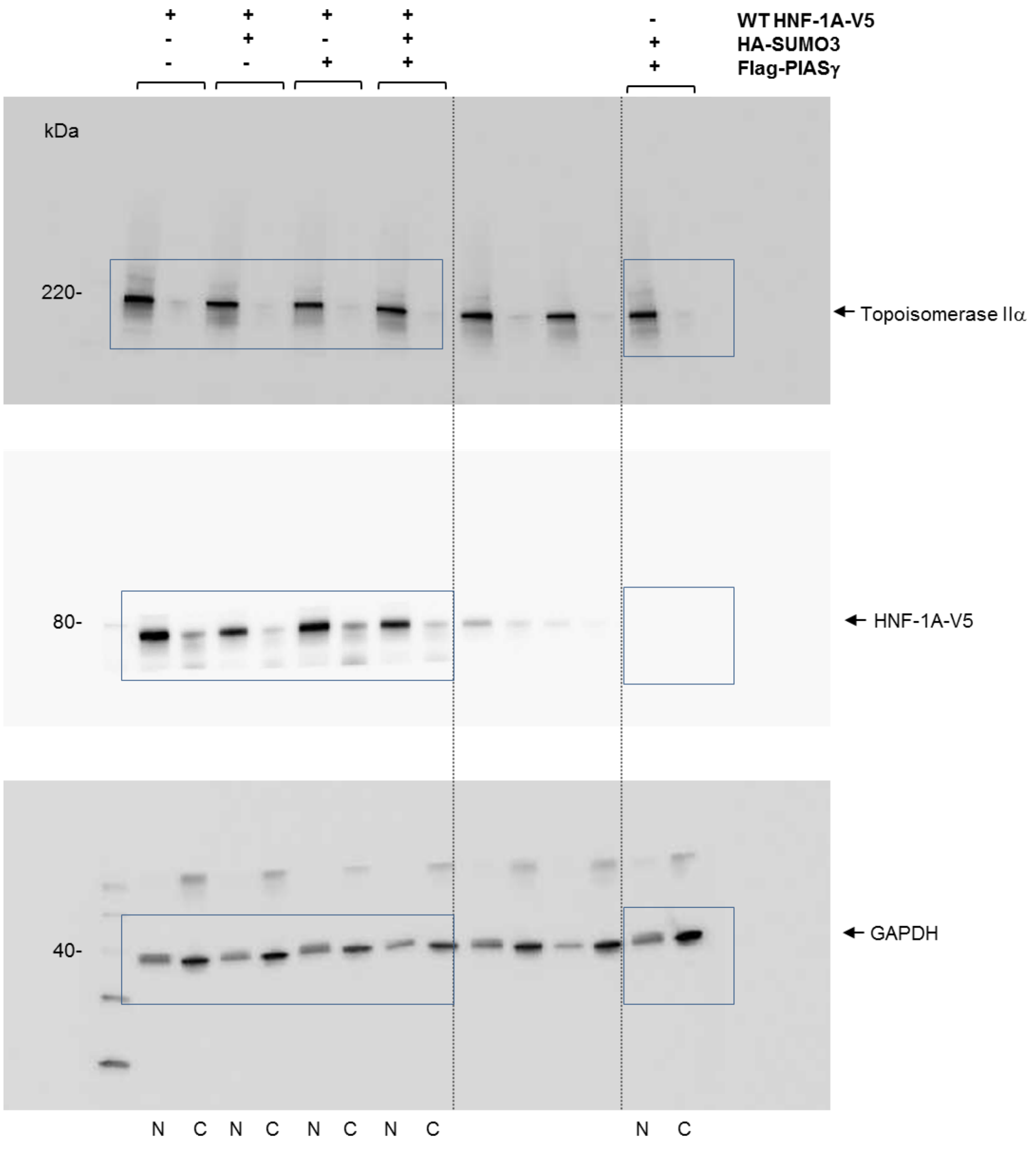

Figure S8: Full scans of blots from main text Figure 6.

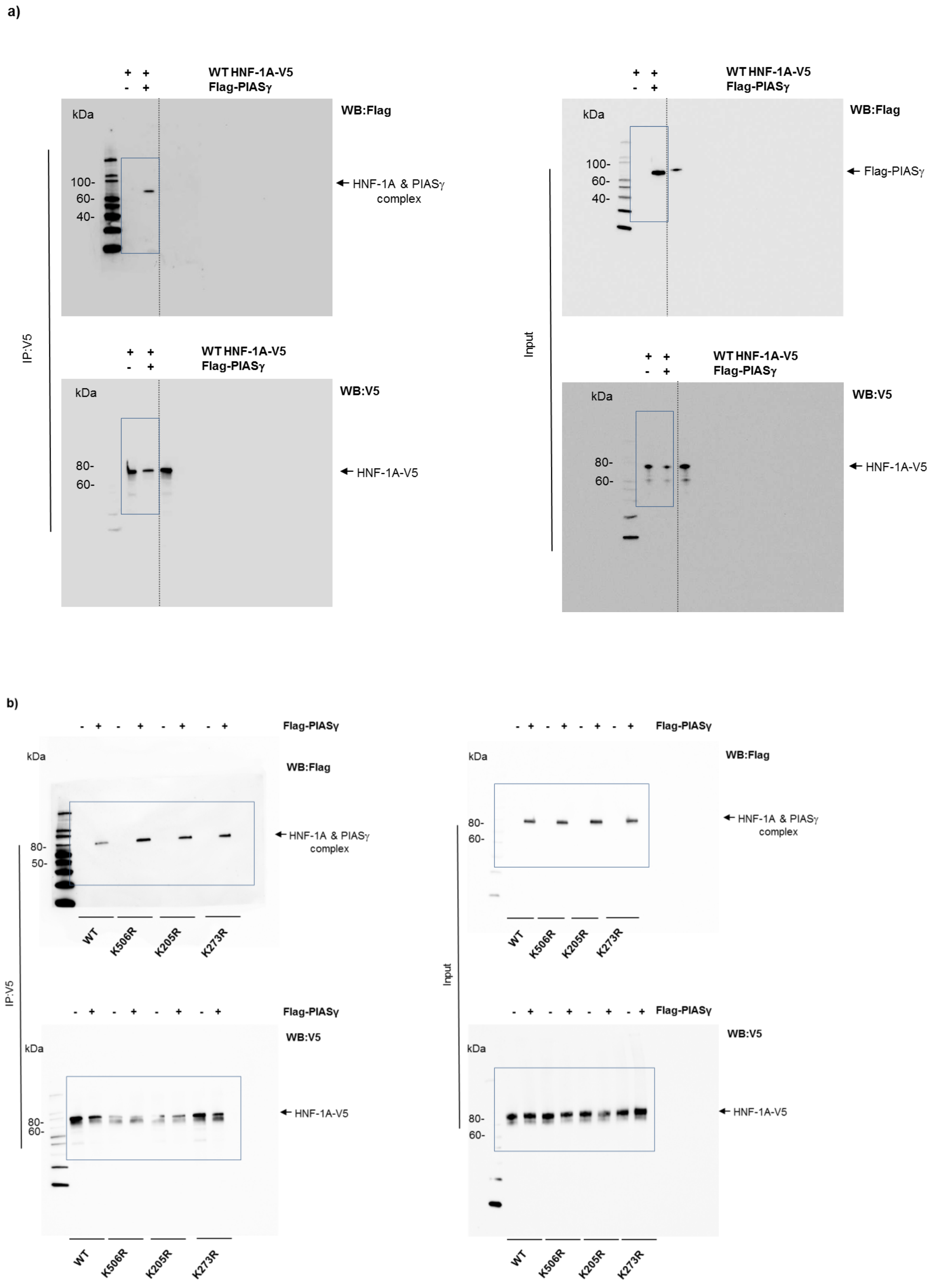

Figure S9: Full scans of blots from main text Figure 7a.

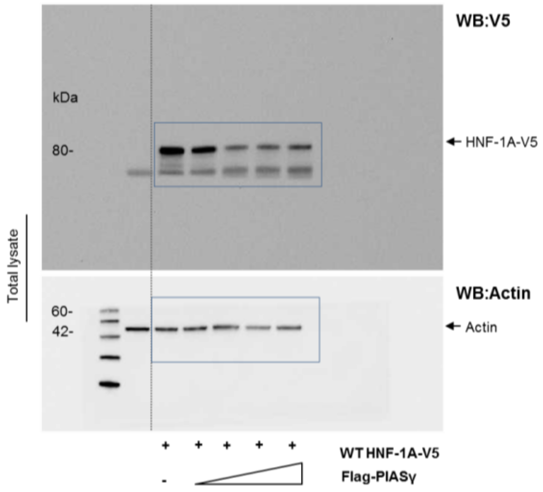

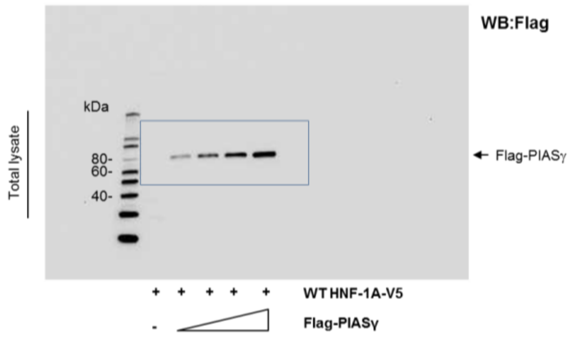

Figure S10: Full scans of blots from main text Figure 7c.

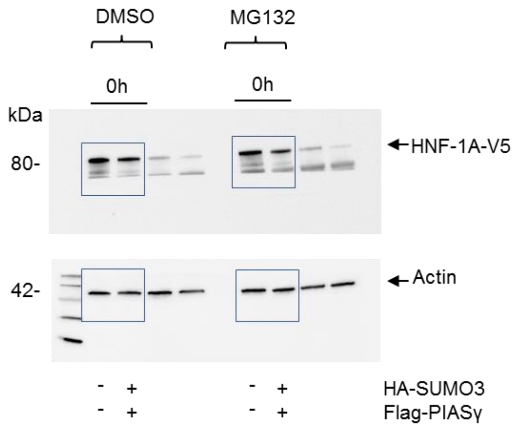

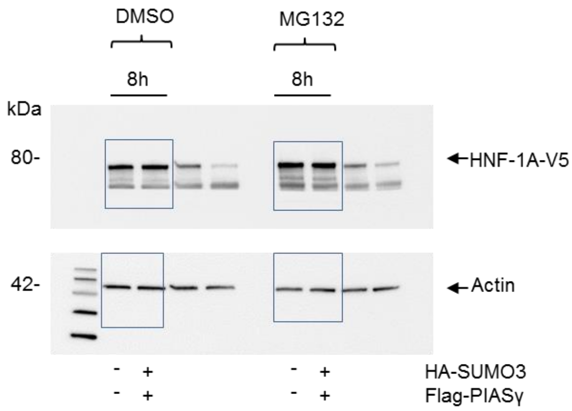

Figure S11: Full scan blots from main text Figure 8a.

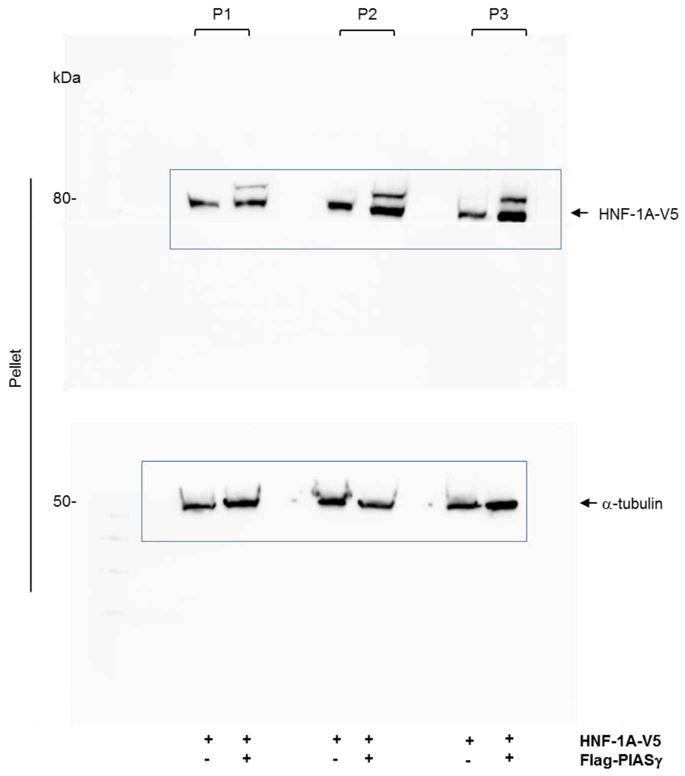

Supplement: Supplementary file 1 — Supplementary information [file 41598_2018_29448_MOESM1_ESM.pdf]
